# Sensitivity to Vocalization Pitch in the Caudal Auditory Cortex of the Marmoset: Comparison of Core and Belt Areas

**DOI:** 10.3389/fnsys.2019.00005

**Published:** 2019-02-01

**Authors:** Shuyu Zhu, Benjamin Allitt, Anil Samuel, Leo Lui, Marcello G. P. Rosa, Ramesh Rajan

**Affiliations:** ^1^Biomedicine Discovery Institute and Department of Physiology, Monash University, Clayton, VIC, Australia; ^2^Australian Research Council, Centre of Excellence in Integrative Brain Function, Clayton, VIC, Australia

**Keywords:** non-human primate, vocalization pitch representation, caudal auditory cortex, spike-count code, first spike-latency code

## Abstract

Based on anatomical connectivity and basic response characteristics, primate auditory cortex is divided into a central core surrounded by belt and parabelt regions. The encoding of pitch, a prototypical element of sound identity, has been studied in primary auditory cortex (A1) but little is known about how it is encoded and represented beyond A1. The caudal auditory belt and parabelt cortical fields process spatial information but also contain information on non-spatial aspects of sounds. In this study, we examined neuronal responses in these areas to pitch-varied marmoset vocalizations, to derive the consequent representation of pitch in these regions and the potential underlying mechanisms, to compare to the encoding and representation of pitch of the same sounds in A1. With respect to response patterns to the vocalizations, neurons in caudal medial belt (CM) showed similar short-latency and short-duration response patterns to A1, but caudal lateral belt (CL) neurons at the same hierarchical level and caudal parabelt (CPB) neurons at a higher hierarchical level showed delayed or much delayed response onset and prolonged response durations. With respect to encoding of pitch, neurons in all cortical fields showed sensitivity to variations in the vocalization pitch either through modulation of spike-count or of first spike-latency. The utility of the encoding mechanism differed between fields: pitch sensitivity was reliably represented by spike-count variations in A1 and CM, while first spike-latency variation was better for encoding pitch in CL and CPB. In summary, our data show that (a) the traditionally-defined belt area CM is functionally very similar to A1 with respect to the representation and encoding of complex naturalistic sounds, (b) the CL belt area, at the same hierarchical level as CM, and the CPB area, at a higher hierarchical level, have very different response patterns and appear to use different pitch-encoding mechanisms, and (c) caudal auditory fields, proposed to be specialized for encoding spatial location, can also contain robust representations of sound identity.

## Introduction

Primate auditory cortex is classically divided into three major, putatively-sequential, hierarchical processing stages, the core, belt, and parabelt regions (Kaas and Hackett, 2000), each with multiple areas likely to have different functions (Tian et al., 2001; Bendor and Wang, 2008; Fukushima et al., 2014). The different areas are differentiated by thalamo-cortical and cortico-cortical connections, tonotopic organization, and neuronal response patterns to sounds (Kaas and Hackett, 2000; Schreiner and Winer, 2007; Romanski and Averbeck, 2009; Hackett, 2015). Differences in response properties can speak to functional specializations of the different areas (Eggermont, 2001; Hackett, 2011) and several studies have demonstrated differences in temporal and spatial response properties of the core and the caudal medial belt (CM) areas (Barbour and Wang, 2003; Bendor and Wang, 2005; Kajikawa et al., 2005, 2008). Characteristics of the pure tone-derived frequency response areas (FRAs) are the main difference reported between auditory cortical areas in primates and other mammals, with bandwidth (Imaizumi et al., 2004; Bizley et al., 2005; Hackett, 2011) and response latency (Bizley et al., 2005; Bendor and Wang, 2008; Hackett, 2011) increasing between core, belt and parabelt areas in line with a cortical hierarchy along this axis.

In the present study we examine the encoding of the pitch of complex vocalizations in primary core field, caudal belt (CB) fields and caudal parabelt (CPB) field in the marmoset monkey, a New World monkey species increasingly used in auditory physiology (Bendor and Wang, 2005; Slee and Young, 2010; Lui et al., 2015; Toarmino et al., 2017; Zhu et al., 2019). The marmoset uses vocalizations in many social behaviors (Epple, 1968; Stevenson and Poole, 1976; Bezerra and Souto, 2008), and marmoset vocalizations differ in spectral composition in ways that allow identification of individuals (Agamaite et al., 2015). A specialized pitch center has been identified in the marmoset auditory cortex in the region of representation of low frequencies (low characteristic frequencies, CFs) that interfaces primary auditory (A1) and Rostral (R) core fields (Bendor and Wang, 2005; Bendor et al., 2012). However, these and other studies of pitch-encoding in primate cortex predominantly used periodic/harmonic complex sounds or artificial vowels with low fundamental frequency (*f*_0_), whereas natural sounds with rich, aperiodic frequency content also can commonly evoke pitch sensations (Yost, 1996; Schnupp et al., 2011). In a recent study of marmoset A1 (Zhu et al., 2019), we found that the majority of neurons in the region of representation of high frequencies (high CF region) can encode the pitch of naturalistic vocalizations, suggesting that distributed activity across A1 can represent the pitch of natural sounds over a functionally-relevant range for differentiating individuals by their vocalizations (Weiss et al., 2001; Bezerra and Souto, 2008).

Different belt regions have been associated with different functions, with CB areas hypothesized to be involved in spatial processing and sound localization (Tian et al., 2001), in line with the dual processing stream theory of auditory perception (Bizley and Cohen, 2013). Certainly, caudal auditory cortex shows specializations to represent sound location (Tian et al., 2001). However it also represents some aspects of sound identity (Recanzone, 2008): neuronal responses of these areas are also sensitive to conspecific vocalizations (Tian et al., 2001; Recanzone, 2008) and human imaging studies have also suggested that the auditory “dorsal stream” areas show activity related to sound identity, as well as location (Giordano et al., 2013; Zundorf et al., 2013).

Little is known of the encoding of pitch, a prototypical sound identity element, in caudal auditory cortex. A hierarchical model whereby individual voices are processed through a neural circuit that also involves projections from the superior temporal cortex to the ventrolateral prefrontal cortex (Romanski et al., 1999, 2005) would predict that non-core auditory fields can encode fine variations of vocalization pitch. However, the FRAs in the non-core areas are broad (Recanzone, 2008) and even in A1, broad response areas have absent or significantly diminished surround inhibition, and reduced neuronal selectivity to broad band stimuli (Suga, 1995; Brosch and Schreiner, 1997; Rajan, 1998, 2001) like vocalizations. Given this, and the fact that A1 neurons exhibit sensitivity to the pitch of vocalizations via mechanisms other than FRA-based frequency-level response (Zhu et al., 2019), there are reasons to suspect that non-core auditory fields may not encode fine variations of vocalization pitch as can A1 (Zhu et al., 2019).

We have now investigated neural responses in A1 and caudal auditory areas to naturalistic vocalizations, and their ability to represent the pitch, a critical sound feature of vocalizations. We found that subpopulations of neurons in caudal auditory cortex exhibited both similarities and difference in temporal response patterns, compared to A1 neurons. Furthermore, neurons in both A1 and caudal areas are able to represent vocalization pitch changes via both response intensity (spike-count) and latency, with different populations showing different coding strategies.

## Materials and Methods

### Animal Preparation

Three adult marmosets (*Callithrix jacchus*, 2 females, 1 male) were used to obtain the data here. All procedures conformed to the guidelines of the Australian Code of Practice for the Care and Use of Animals for Scientific Purposes, and were reviewed and approved by the Monash University Animal Experimentation Ethics Committee.

Surgical procedures have been detailed in previous auditory cortex studies (Rajan et al., 2013) and in studies of visual (Lui et al., 2006) and motor (Burman et al., 2008) cortex physiology. The marmoset was pre-medicated with intramuscular injections of diazepam (3 mg/kg) and atropine sulfate (0.2 mg/kg), and anesthetized, 30 min later, by intramuscular injection of Alfaxan (10 mg/kg; Jurox, Rutherford, Australia). When deep anesthesia was established (confirmed by absent withdrawal reflexes to noxious forepaw pinching), the animal was placed on a heating blanket, tracheostomized, and had a femoral vein cannulated. It was transferred to a surgical table in a sound-attenuated room, and placed on a heating pad with a rectal probe to maintain body temperature, through feedback control, at 37°C. Surgery was conducted to expose the skull and to expose and transect the external auditory meatuses bilaterally so that the eardrums could be visualized. The head was stabilized by fixing the forehead to a head bar in a magnetic stand, using a screw and dental cement to anchor the head bar on the skull. The left auditory cortex was exposed by craniotomy and the dura removed; the cortex was kept moist and clean with warm saline throughout the experiment. The animal was then switched to maintenance anesthesia with intravenous infusion of sufentanil (8 μg/kg/h; Janssen-Cilag, Sydney, Australia) with dexamethasone (0.4 mg/kg/h; David Bull, Melbourne, Australia) diluted in Hartmann's solution (injection volume 1.5 ml/h). Artificial ventilation was introduced with nitrous oxide and oxygen (7:3) delivered via the tracheal cannula.

These procedures and the anesthetic regime have been shown to have minimum effects on neuronal activities in marmoset auditory cortex to both simple and complex stimuli (Rajan et al., 2013), while maintaining the animal in an anesthetized state (Burman et al., 2008).

### Electrophysiological Recordings

Recordings were made using a 32-contact single-shank linear electrode array with vertical inter-electrode spacing of 50 μm (A1x32; NeuroNexus, Ann Arbor, MI, USA). Amplification (× 1,000) and filtering (bandpass 750 Hz−5 kHz) of the electrode signal were done using TDT Systems Model RA4PA (Tucker Davis Technologies, Alachua, FL, USA) Medusa Pre-amplifier and a Cereplex Direct (Blackrock Microsystems, Salt Lake City, UT, USA) station. Spiking activity was detected by manually setting the spike-amplitude threshold during recording. Spiking history and raw spike waveforms were stored for online and offline analysis. Spike sorting was performed using principal component analysis (PCA) on waveforms recorded from each electrode. Clusters of spikes were identified by fitting a mixture of Gaussians to the PCA space of the normalized waveform (Jain et al., 2000; Ghodrati et al., 2016). Single-units were identified where the recorded waveform had a signal to noise ratio (SNR) >2.75; otherwise, recordings were classed as multi-units (Kelly et al., 2007; Smith and Kohn, 2008).

Recordings were obtained from seven auditory cortical fields: three core fields [primary auditory cortex (A1), rostral field (R), and rostral temporal field (RT)], three belt fields medial lateral belt (ML), caudal belt (CB; including caudomedial [CM] and caudolateral [CL] fields), and the CPB field. Allocation of penetrations to these fields was done principally on the basis of the reliable changes in tonotopic sequence across auditory cortex (as also seen in other animals not part of this study), and on the physiological response characteristics (e.g., FRA bandwidth, response latency, response duration). Additionally, in some cases, comparisons of the histology of auditory cortex were made to our other work (Majka et al., 2018). To ensure we obtained good quality data for all fields, we recorded from no more than 3 fields in any animal. In all animals, we always recorded data from A1 and A1 recordings were obtained at multiple stages during the course of the 3–4 day experiment. This protocol allowed us to have a cross-animal comparator and only if data in A1 were stable within an animal and were comparable between animals, did we then also use data from other fields that may or may not have been common across the animals. This protocol gives us confidence in comparing between fields even if the data from some non-A1 fields were from different animals; in fact, based on this protocol we excluded from this report data from a fourth animal in which recordings were made in A1 and CM, as the A1 data were aberrant compared to A1 data from all other animals. In this report, we present data for A1 and the caudal belt only from animals in which the A1 data were not significantly different from each other at the population level and the A1 data from an individual animal was stable across time; note that the A1 spike-count data were reported previously (Zhu et al., 2019).

Here we investigate the representation of pitch, a prototypical sound feature, in A1 and in the non-core fields CM, CL, and CPB, thought to be specialized for sound localization (Tian et al., 2001). Multiple penetrations were made in each animal along the lateral bank of the lateral sulcus and superior temporal surface, to obtain recordings from different auditory fields. Across all animals, we obtained 292 responsive units (pooled multi-units and single-units; see section Data Analysis below) in A1 and 629 responsive units in caudal non-A1 areas: from animal 1,504, 79 (27%) units in A1, 82 (98%) units in CM, and 12 (4%) units in CL; from animal 1,512, 81 (28%) units in A1, 2 (2%) units in CM, 278 (90%) units in CL and 237 (100%) in CPB; and from animal 1,598, 132 (45%) units in A1 and 18 (6%) units in CL; thus, apart from CPB, data for all other fields were obtained from at least two animals.

### Stimuli

Stimulus presentation was done using custom-written programs in MATLAB (MathWorks, Natick, MA). Pure tone stimuli were generated by a TDT RX6 multifunction processor (Tucker Davis Technologies), which was also used for presenting vocalization call stimuli stored as “.wav” files. The vocalization calls were the same as in previous studies (Lui et al., 2015; Zhu et al., 2019). The pure tone stimuli and vocalization calls were passed to a PA5 programmable attenuator and thence to an HB7 headphone driver (Tucker Davis Technologies), before delivery via MF1 multi-function speakers (Tucker Davis Technologies). To calibrate the stimuli, the sound from the speaker was captured by a condenser microphone placed in a closed sound coupler to mimic the marmoset external ear, at 1 mm from the sound delivery tube, and amplified using a type 2,673 microphone, powered by a type 2,804 microphone power supply. Frequency response calibration curves of each speaker were generated to calibrate the system output to present stimuli at desired Sound Pressure Levels (SPLs). During an experiment, the speaker delivery tubes were fitted in the external ear canal and inserted to about 1 mm from the eardrum.

**Figure 1:**
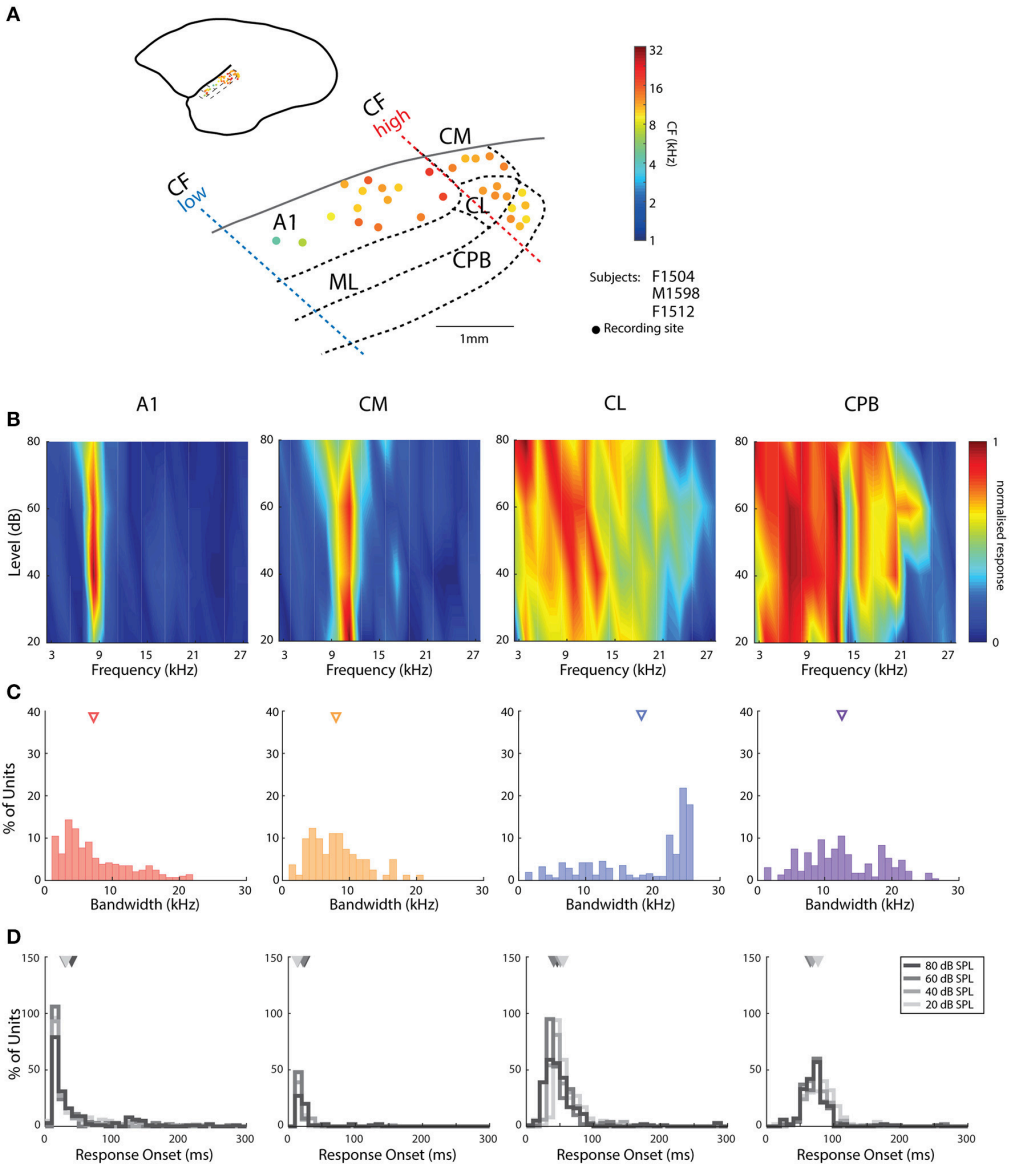
Cortical recording sites, located in different cortical areas, differentiated by response characteristics. **(A)** Map of the spatial distribution of Characteristic Frequency (CF; frequency of lowest threshold) of neurons in the different auditory areas demarcated by bold dashed lines (see text for details for differentiation between fields). Filled dots indicate responsive recording sites with color indicating the mean CFs (see color map to the right of the figure) across all recording electrodes in the penetration whose location is indicated by the placement of the dot relative to the lateral sulcus (LS) and surface vasculature. **(B)** Representative frequency response areas (FRAs) from a neuron in each area (denoted above each column), with responses normalized to the maximum response in the FRA (see color map). **(C)** Distribution of FRA bandwidths of neurons in the different cortical areas; triangles mark the mean bandwidth of each distribution. **(D)** Distribution of response onset to CF at 20, 40 60, and 80 dB SPL; different color indicates distribution at different SPL; triangles represent the mean of the distribution. A1, primary auditory cortex; CM, caudal medial belt; CL, caudal lateral belt; ML, medial lateral belt; CPB, caudal parabelt (CPB).

To characterize the frequency response space of individual units and to determine the locations of recording sites based on auditory cortex tonotopy (see Figure 1), pure tone stimuli were presented to the ear contralateral to the recording left hemisphere to obtain FRAs. The FRAs were determined by presenting pure tones at 18 linearly spaced frequencies from 3 to 28.5 kHz in 1.5 kHz steps, at levels from 20 to 80 dB Sound Pressure Level (SPL) in 20 dB steps, with the entire matrix presented 10 times. Frequency-level combinations were randomly presented as 100 ms tone bursts with 0.5 ms cosine rise-fall ramps, with 500 ms inter-stimulus interval. The responses to each frequency-level combination were summed online and displayed as tone FRAs.

We examined the responses of auditory cortical neurons to variations in the dominant pitch (*f*_*dom*_; see Agamaite et al., 2015) for four marmoset vocalizations (“Egg,” “Ock,” “Tsik,” and “Twitter”). The calls carry different social-behavioral meanings. “Egg,” “Ock,” and “Tsik” are social mobbing calls with the first associated with vigilance behavior and the latter two used to attract and locate conspecifics, and “Twitter” is used at visual contact with conspecifics (Epple, 1968; Stevenson and Poole, 1976; Bezerra and Souto, 2008). Only the first syllable of each call was used to keep stimulus duration similar (35, 42, 68, and 66 ms for Egg, Ock, Tsik, and Twitter, respectively). The calls vary spectrally in both dominant frequency and harmonic structures. “Twitter” and “Tsik” calls have similar frequency components, with dominant frequencies around 8–10 kHz, and strong first harmonics around 20 kHz. “Tsik” also has a second harmonics at up to 30 kHz. In contrast, the “Ock” and “Egg” calls have very different frequency content, with most power below 1 kHz.

All calls were resampled at 196 kHz and stored as.wav files. For each call, up to 13 pitch-shifted calls were generated, with calls being shifted from the original signal by 6, 4, 3, 2, 1, and 0.5 semitones up and down in pitch, using the Phase Vocoder method (Gotzen et al., 2000). All calls and pitch-shifted tokens were calibrated to give an average bilateral level of 70 dB through the TDT system. In each penetration, we tested each call with all pitch tokens presented in random fashion, until each stimulus was presented at least 30 times. The order of testing of calls was randomized, and trials were interleaved with 1 s inter-trial interval. Only data from penetrations where all 4 calls were tested were analyzed.

### Data Analysis

To investigate population coding of vocalization pitch in auditory cortical fields, we analyzed single and multi-unit data and found similar patterns in both types of neuronal recordings. Therefore, we will report the pooled single and multi-unit data, referred to as *units* throughout this report. Data analysis was performed using custom-written scripts in MATLAB. For some penetrations, only 11 pitch variations were tested. Therefore, in information theory analysis and pitch discriminability analysis, only data from the commonly tested 11 pitch variations were used (−4 to +4 semitone shifts).

### Characterization of Response Pattern

Pure tone stimuli were used to characterize each unit's responses in frequency-intensity space (the frequency-response area, FRA), with spike rate at each pure tone frequency-intensity combination calculated by averaging spike-count in a 100 ms window after stimulus onset. From the FRA, we determined the unit's Characteristic Frequency (CF) as the frequency which evoked the highest response at the lowest intensity. At each test SPL, unit tuning bandwidth was determined by finding the frequency range that reliably evoked a response >2 SD higher than spontaneous activity, which was defined as the mean firing rate over 200 ms prior to stimulus onset. Linear interpolation was performed between the threshold intensities at each test frequency to generate the bandwidth at 70 dB SPL, the SPL at which vocalizations were presented.

To define differences between units and fields in the temporal response patterns to pitch tokens, for each pitch token in each unit we generated peri-stimulus time histograms (PSTHs) representing the mean spike-count in 1 ms bins across all trials for that pitch token and response onset was taken as the first bin of 5 consecutive bins with a firing rate that exceed the 95% confidence interval of the spontaneous firing rate. Mean response onset was calculated across all pitch tokens for each call. To compare the consistency of response onset between calls, response onset time from all units of different populations were pooled. Comparisons were made between all possible combinations of calls (4 calls tested, 6 different combinations of pairs of calls). The population response onset between pairs of calls were fitted using linear regression. The slope of the fitted regression line was compared to a slope 1.

The population response temporal pattern to each call was determined (see **Figure 4**) by first normalizing the PSTH, of individual responsive units, to each pitch token of that call (to minimize the effects of pitch response on temporal patterns), then averaging the normalized PSTHs across all pitch tokens to generate the mean response of that unit to the given call, and then averaging the mean PSTHs of all responsive units to generate the population response temporal pattern. From the mean PSTH of each unit to a given call, response duration was also calculated as the half-peak width of the mean PSTH, i.e., the time span when responses were ≥50% of the normalized response. For responses with two clearly-defined peaks (e.g., in CL, **Figure 4**), the half-peak width was calculated about each peak and summed for a single value.

To assess tuning to pitch variations of each vocalization, we applied two metrics to responses to each token: the first spike-latency to the token (Bizley et al., 2010), and the mean spike-count (Zhu et al., 2019). To calculate first spike-latency, the raw spiking data was used without correction for spontaneous activity. First spike-latency was determined as the time of the first spike observed after stimulus onset. Mean spike-count was calculated by averaging the spike-count of a 40 ms window, whose start time was set individually for each pitch token to account for any variation in spike-latency as follows: for each pitch token, the 25th percentile of first spike-latency distribution across trials was determined, and the start of the spike-counting window was then set to be 5 ms before that value for the first spike-latency (see **Figure 5**). The use of a moving window for counting spikes avoids potential confusion of latency tuning with spike-count tuning, which could be introduced by inappropriate choice of counting window start and length.

For all cortical fields, we used a 40 ms window, so that only the onset response to the stimulus was captured. Both latency tuning and spike-count tuning to pitch was assessed by one-way ANOVA and units determined to be tuned to pitch changes if their spike-latency or spike-count was modulated in a statistically significantly manner by pitch variations.

### Information Theory Analysis

To assess information about pitch contained in the spike-count and first spike-latency, we computed the amount of information using a method that calculated the conditioned entropy (Cover and Thomas, 2006; Quian Quiroga and Panzeri, 2009) which quantifies the reduction of the observers' uncertainty about the pitch of the stimulus with the knowledge of the response measured either in spike-count or first spike-latency:

(1) $$\begin{array}{l} I_{count/time}=\sum_{k=1}^{S}P\left(k\right)\sum_{r=1}^{r_{\max}}P\left(r|k\right)\log_{2}\frac{P\left(r|k\right)}{P\left(r\right)}, \end{array}$$

where *r* represents either the spike-count or first spike-latency to the stimulus, *k* represents the stimulus condition and *S* represents the total number of stimuli. To minimize bias caused by binning, the number of bins was set to be the same for I_count_ and I_time_ calculations to match the response categories for spike-count and first spike-latency. In ideal cases where the stimulus representation was perfect, the maximum bits of information with 11 equal probability stimulus conditions is 3.5 [log_2_ (11)].

We also computed information content using Victor's binless method (Victor, 2002), which controls the bias introduced by binning responses (Chase and Young, 2007). To compute information in first spike timing (I_time_), we applied the Chase and Young (2007) equation:

(2) $$\begin{array}{l} I_{time}=\;\frac{1}{N}\sum_{j=1}^{N}\left(\frac{\lambda_{j}}{\lambda_{j}^{{\;}^{*}}}\right)-\sum_{k=1}^{S}\frac{N_{k}}{N}\log_{2}\frac{N_{k}-1}{N-1}, \end{array}$$

where *N* represents the total number of stimulus presentations; *N*_*k*_ represents the total number of presentations of the *k*th stimulus; *S* represents the total number of stimuli used; *j* represents the first-spike samples and is projected onto an n-D space, with the coordinates of the spike samples determined by the *nth* Legendre polynomials where *n* corresponds to the total number of spikes in the spike train of a trial. Since we only consider the information in the first spike time, we set *n* = 1. These embedding coordinates are then used to calculate the Euclidean distance between spikes. λ_*j*_ represents the minimum Euclidean distance between spike sample *j* and all other first spikes, and $\lambda_{j}^{*}$ represents the minimum Euclidean distance between spike sample *j* and other first spikes elicited by the same stimulus. I_time_ represents the reduced uncertainty in spiking pattern when spike time is taken into consideration.

Information in the spike-count (I_count_) was computed with Equations (15) and (16) in Victor (2002):

(3) $$\begin{array}{l} I_{count}=\;-\sum_{n=0}^{n_{\max}}\sum_{k=1}^{S}\frac{N\left(n,a_{k}\right)}{N}\log_{2}N\left(n,a_{k}\right)\\ \;+\sum_{n=0}^{n_{\max}}\frac{N\left(n\right)}{N}\log_{2}N\left(n\right)+\sum_{k=1}^{S}\frac{1}{N_{k}}\log_{2}\frac{1}{N_{k}}\\ \;+\frac{\left(S-1)(n_{\max}-1\right)}{2Nln2}, \end{array}$$

where *N(n,a*_*k*_*)* is the number of trials in which a stimulus *a*_*k*_ elicited *n* spikes; *n*_*max*_ is the maximum spike-count elicited by any stimulus; and *N*_*k*_ represents the number of stimuli used. In this method, the entropy of spike trains that elicited the same number of spikes was calculated separately. The last term in Equation (3) represents the classical correction for entropy estimates. I_count_ represents the reduced uncertainty in spike pattern when the spike-count that elicited by given stimulus is known.

### Pitch Discriminability

We assessed pitch discriminability of units by calculating the area under the receiver operating characteristic curve (ROC) between the two extreme conditions in the spike-count tuning curve or the latency tuning curve, viz., between pitch of peak response and pitch of lowest response in the respective tuning curve (Britten et al., 1992; Zhu et al., 2019). For spike-count, the preferred pitch for a call was the token with the highest spike-count and null pitch that with the lowest. For first spike-latency, preferred pitch had the shortest mean first spike-latency and null pitch had the longest.

We tested population pitch discriminability in each field using a linear decoder, using a multiclass support vector machine (SVM) to generate the probability for each of the pitch variation of a given call based on the distribution of the response (Duda et al., 2001). To compare the performance of the spike-count-based decoder with the first spike-latency-based decoder, we sampled a portion of units tuned to pitch changes by either measure for integration windows of 1, 2, 4, 6, 8, 16, 32, 64, 128, 256, 512 ms. With each integration window, we accumulated spikes from the onset of responses up to the end of the window. We trained each decoder with 80% of the trials randomly selected from each unit and tested with the remaining 20% of the trials from that unit, to determine the predictability of the call identity. Decoder performance for the prediction of call identity was calculated as the percentage correct identification of the test trials. Variability of decoder performance was estimated by decoding 20 different subpopulations or repeat the decoding procedure for the entire population but with 20 different sets of training and testing trials.

### Statistical Analysis

As described above, a variety of different types of statistical analyses was used to compare various data between the different cortical fields. For overview, the tests and their use are summarized here; the use of each test is detailed appropriately in the Results. For all analyses, the significance level was always set to 0.05.

- Comparisons between cortical fields of bandwidth, response duration, and information content were done using the Kruskal–Wallis test with Dunn's multiple comparisons test because the D'Agostino & Pearson normality test showed that these population data were not normally distributed.
- Tuning of spike-count/latency to pitch variations for each call was examined using one-way ANOVA with Tukey's HSD test since normality was confirmed for these data using the D'Agostino & Pearson normality test.
- In each field, the relationship between response onset times between calls was determined using linear regression analyses. Here, the response onset time for a call from all units of that field were pooled and then comparisons were made between all possible combinations of calls using linear regression, with the slope of the fitted regression line compared to a slope of 1.
- The distributions of the proportion of units pitch tuned to each call by spike-count or spike-latency measures were compared between the different cortical fields using Chi square tests.
- Pitch discriminability of units was determined by calculating the area under the receiver operating characteristic curve (ROC). Two-way ANOVA was used to compare aROCs for each call with either measure within a given cortical field. Sidak's multiple comparisons test used to test for the effects of different coding measures for on mean aROC for each call separately.
- Population pitch discriminability was assessed by decoder analysis. Two-way ANOVA was used to compare decoder performance between cortical fields and across integration windows. Since D'Agostino & Pearson normality test confirmed the data was normally distributed, Dunnett's multiple comparisons test was used to examine performance changes with changes in size of the integration window. One-way ANOVA was used to examine the effect of changes in sample size on performance within each cortical field for each call separately. For the one-way ANOVA, we subsequently did not perform multiple comparisons to see which of the sample sizes showed significantly different performance compared to others since we were only interested in the overall change of decoder performance with sample size.

## Results

We obtained recordings of neuronal responses to pure tones and conspecific vocalizations from neurons in primary auditory cortex (A1), the caudal medial belt (CM), and caudal lateral belt (CL) belt areas, and the CPB (Figure 1A). To define the encoding of vocalization pitch, we report data from units responding to at least one pitch token of any call, amounting to 292 A1 units neuronal clusters (including 84 single-units), 84 CM units (including 30 single-units), 308 CL units (including 124 single-units), and 237 CPB units (including 127 single-units). We will refer to these multi-unit clusters as *units* hereafter. Note that the A1 data was that also used in our previous report on pitch coding in A1 (Zhu et al., 2019), but data from all the other fields are fresh to this study.

**Figure 2:**
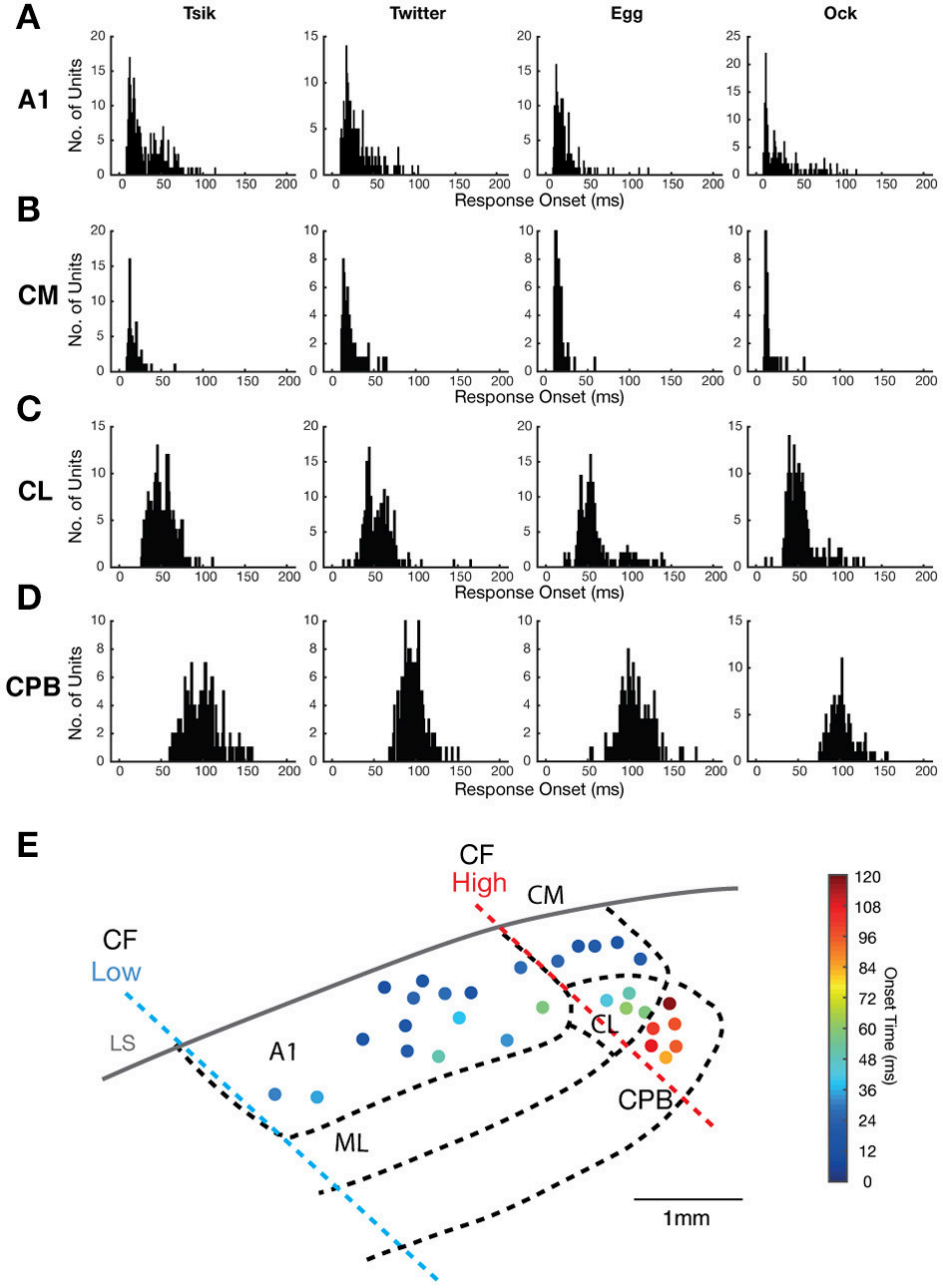
Distribution of neuronal response onsets to vocalizations in the different cortical fields. **(A–D)** Each panel in **(A–D)** shows the distribution of the onset of responses of individual units in a cortical area (see label beside each row) to a particular vocalization (see label above each column). **(E)** A map of the spatial distribution of response onset superimposed on to the map of cortical areas (see Figure 1), with the borders between different auditory areas showing in bold dashed line. Filled dots indicate responsive recording site with the color corresponding to the mean response onset time across the recording electrodes.

Assignment of penetrations to these subdivisions of auditory cortex was based on converging evidence from electrophysiological mapping of responses to pure tones (cochleotopic organization) and histological examination of the electrode tracks. As reported previously in marmoset auditory cortex (Aitkin and Park, 1993; Nelken et al., 1999; Kajikawa et al., 2005; Bendor and Wang, 2008; Mesgarani et al., 2014), we found a cochleotopic low-to-high CF gradient from rostral to caudal A1 (Figure 1A) and a reversal of this gradient in the roughly 1 mm wide region of the caudal belt; penetrations further caudal to CM did not contain units responsive to either tones or vocalizations (Figure 1A), indicating this is likely not part of auditory cortex (Majka et al., 2018). Our results are in line with parcellation into core, belt and parabelt auditory cortical areas on the basis of the tuning characteristics of neurons in sites assigned to these subdivisions (Figures 1B,C) and latency of responses to tones and vocalizations (e.g., Figures 1D, 2). Neurons in CL showed broader tuning to tones than those in CM (Figures 1B,C), and delayed response onsets to vocalizations (Figure 2). Responsive CPB units, located further lateral to CL, also had broad tuning to tones (Figures 1B,C), but were particularly distinguished by their very long latencies in responses to vocalizations (viz., Figure 2).

### Response Characteristics to Pure Tones

The pure tone bandwidths of A1 and CM units had similar distributions (Figure 1C; Kruskal–Wallis test, mean rank diff. = 75.02, *p* = 0.19). The bandwidths in CL were heterogeneous and covered a large range, which may suggest subdivisions within this area, or distinct populations of neurons. Mean bandwidth was widest among CL neurons and was significantly broader than for all other populations [Kruskal–Wallis test, H(4) = 54.11, with Dunn's multiple comparisons test (Dunn's test), *p* always < 0.0001], while mean bandwidth in CPB was also significantly larger than in CM and AL (Dunn's test, *p* always <0.0005). A few units from recording sites lateral to rostral A1 also showed broader tuning curves (data not shown). Additional to bandwidth, response onset to tones was also a good differentiator as it gradually increased from A1 and CM to CL and was longest for CPB units (Figure 1D). Unlike the other fields, we observed that response onset in CL and CPB to tones was modulated by stimulus frequency, as reported previously (Zhou et al., 2012).

### Temporal Patterns of Responses to Vocalizations

As noted, there were clear differences between areas in response latency. To quantify this, which may speak to the issue of hierarchical flow of information from core through belt to parabelt auditory cortex, we calculated the mean response onset across all pitch-varying tokens for each call for each unit. The distribution of this mean response onset for each vocalization is shown in Figure 2 for all units that responded to the four vocalizations. The response onset distribution for all four areas was unimodal, but showed significant differences. The majority of A1 and CM units showed early response onsets to all calls (Figures 2A,B), with mean response onset across calls at ≈29 ms for A1 and 18 ms for CM; the latter field had more tightly clustered mean response onsets for all four calls, and mean response onset times >50 ms were especially rare among CM units. The CL units (Figure 2C) had more delayed response onsets, with mean response onset across calls at ≈54 ms, while the CPB units (Figure 2D) showed even slower response onset latencies, averaging at ≈100 ms across calls. The CPB population was clearly distinguished from that in CL by the much larger number of units with response onset >80 ms for all calls. These patterns of increasing latency from medial to lateral are shown in a cortical surface spatial map of response onset changes to calls (Figure 2E).

**Figure 3:**
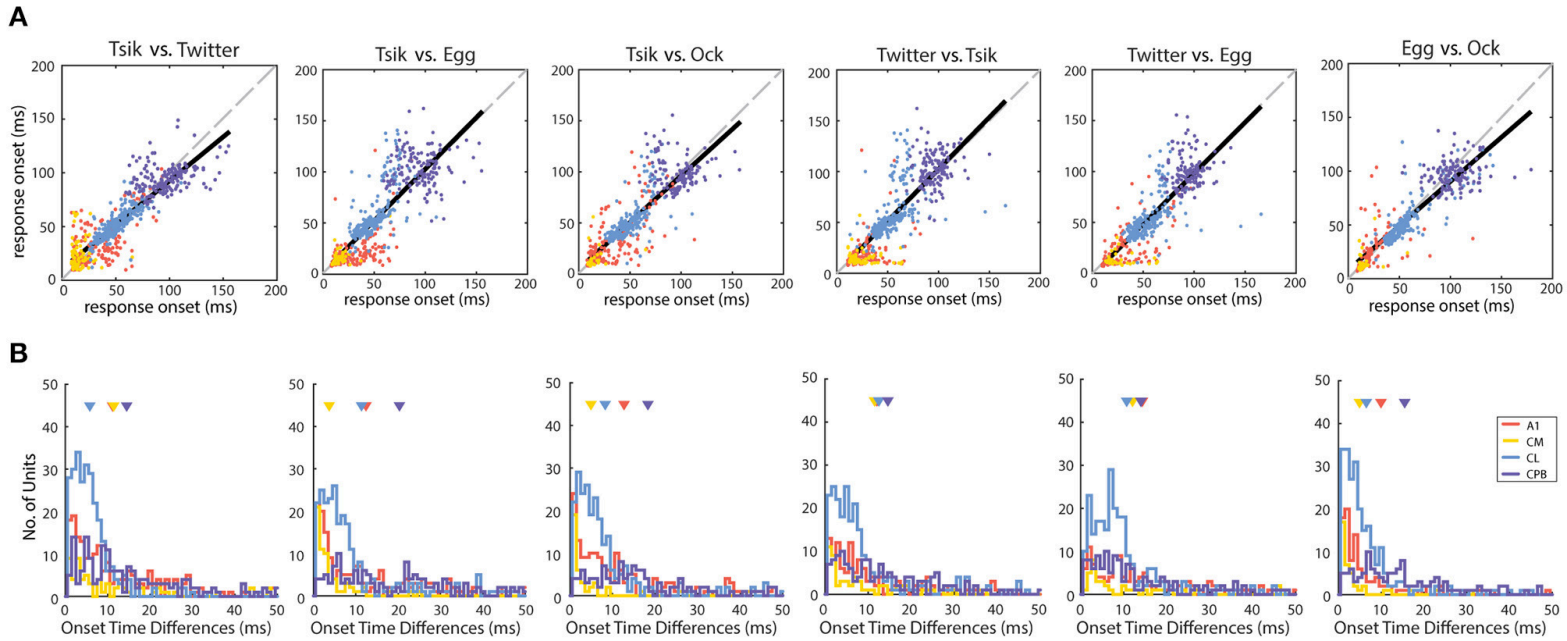
Distribution of response onset time differences between calls in different auditory cortical areas. **(A)** The unit-specific response onset for a pair of calls was plotted against each other (pair being compared is shown above each panel). The color of the dots indicates the population of the units. The solid black line indicates the linear regression line of the response onset. The dotted gray line indicates the line of unity. Note that in most comparisons, the regression line is very close to the line of unity. **(B)** The unit-specific difference in response onset between a pair of calls was calculated for all units that responded to the two calls being compared (pair being compared is shown above each panel). The distributions of these unit-specific differences in response onset time listed are plotted for each of the cortical areas (different colors for the different populations). In each panel the triangles indicate the mean onset time differences for each of the areas. Note that in all comparisons, the great majority of units showed differences of <10 ms between response onsets for any pair of calls, even in cortical areas with later response onsets (CL and CPB).

In all fields, response onset for any one unit remained relatively constant across calls. For each unit that responded to at least two calls we derived a single mean response onset time across all pitch-varied tokens for each specific call, and compared these mean response onsets in individual units between all possible pairs of calls (Figure 3A). Linear regression analysis revealed significant correlations for all pairs of calls (for all pairwise comparisons: *R*^2^ always >0.70 and *p* always <0.0001), indicating a strong correlation of response onset between calls across the entire population. Further, across all comparisons, the slopes of the regression lines did not differ significantly from 1 except for three comparisons-*Tsik* vs. *Twitter, Tsik* vs. *Ock*, and *Egg* vs. *Ock* (*p* always <0.05); note that these exceptions were not from comparison across different classes of vocalization *f*_*dom*_ since *Tsik* and *Twitter* both have similar high *f*_*dom*_ while *Egg* and *Ock* have similar low *f*_*dom*_.

The distributions of differences in response onset time between pairs of calls, for each cortical area, are shown in Figure 3B. In line with the linear regression analysis, for most units, differences between response onset of pairs of calls were <20 ms; for most comparisons between pairs of calls for each cortical field, mean differences were within this range except for CPB units for the comparison between response onsets to *Tsik* and *Egg* (mean onset time differences of 20.3 ms). In summary, for units across all populations, response onset time was closely correlated and maintained across calls.

**Figure 4:**
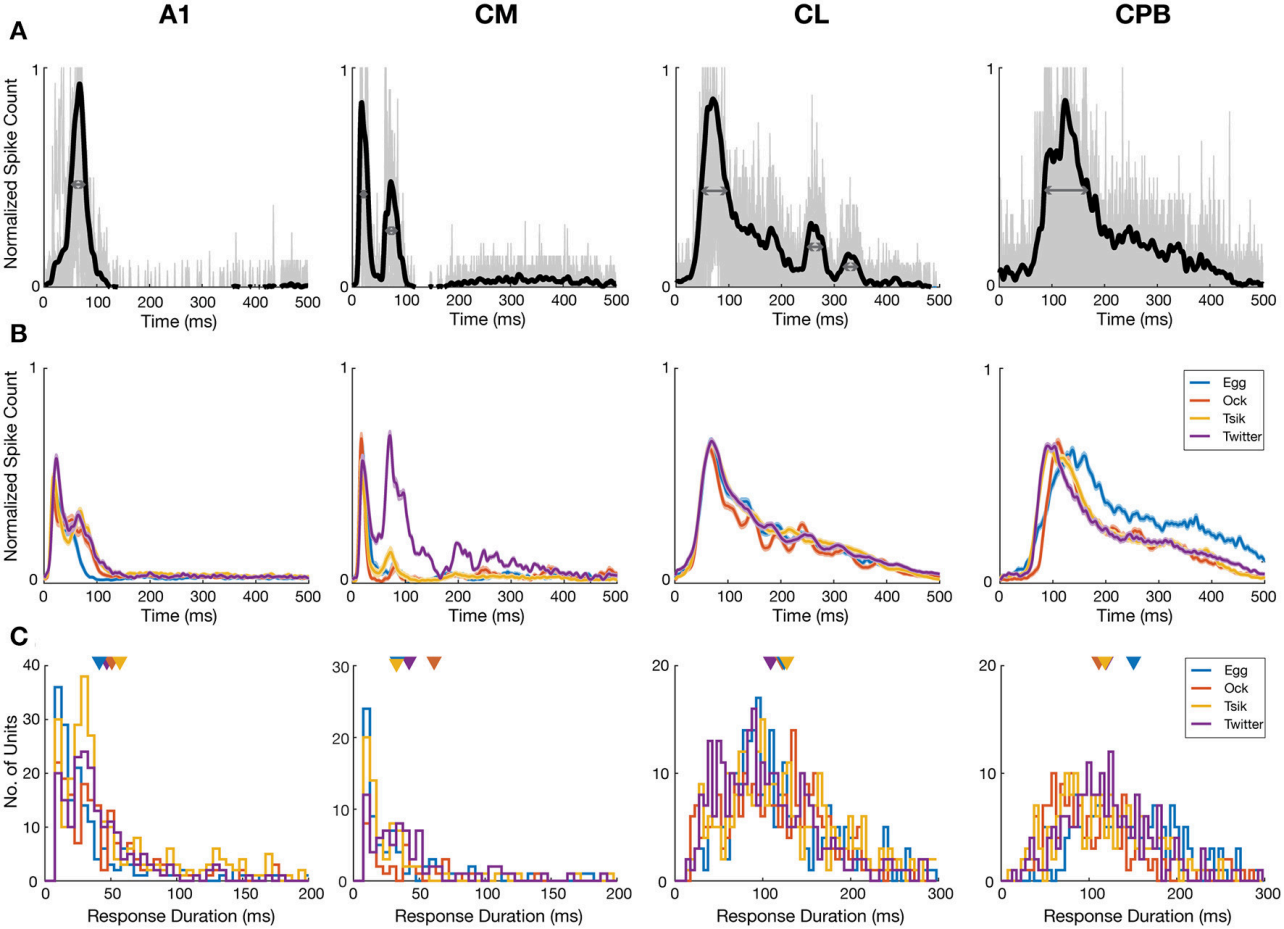
Response temporal patterns and distributions of response duration. **(A)** Example normalized mean PSTHs across pitch tokens and individual responses to different pitch tokens of a given call. Black curve indicates the normalized mean response of a unit in each cortical area. Gray curves indicate the responses to each pitch tokens of a given call. Dark gray arrows indicate the half-peak width of the response which is termed as the response duration of the unit to that call. **(B)** Mean normalized PSTHs for different calls in the different auditory cortical areas A1, CM, CL, and CPB. Each cell's responses are pooled from different pitch tokens of each call, to generate a single normalized PSTH for each unit for each call and then averaging the normalized PSTH for that call across all units in that cortical area to yield a single mean PSTH for that call. Shading indicates standard error of the mean response. Different color indicate response to different calls. Note that in A1 and CM response peaks occurred early, and responses were generally short lasting and complete within ≈100 ms from stimulus onset, whereas in CL and CPB response peaks occurred much later and responses were much longer-lasting, up to 500 ms post-stimulus onset. **(C)** Distributions of response duration, calculated as the half-peak width of the PSTH, of individual units to different calls. Color indicates different call and triangle indicates the mean of the distribution.

The four cortical fields differed in temporal response patterns (Figure 2) beyond response onset time. For each unit, we first normalized the unit's PSTHs of each pitch token of each call. These normalized PSTHs were then averaged to generate the mean PSTH of the unit to that call, which was then itself normalized to a maximum of 1. We pooled all the call-specific normalized mean PSTHs from all responsive units in each cortical area and calculated the mean population PSTH for each call (Figure 4B). Both A1 and CM had short lasting responses across all four calls, with early response peaks-well before ≈40 ms and responses generally confined to 100 ms from stimulus onset. The A1 patterns were very consistent across calls with a response peak at ≈25 ms and another at ≈80 ms. In CM all four calls elicited the same tight response peak at ≈20 ms, and three calls elicited a much smaller second peak at ≈90 ms from stimulus onset; the second peak, to the *Twitter* call, at ≈90 ms, was much larger and, at the mean population level, even larger than the first peak. The other two fields, CL and CPB, had a single, later response peak to all calls, at ≈90 ms from stimulus onset for CL and ≈110 ms for CPB; in both fields, responses declined gradually thereafter, especially in CPB, up to 500 ms post-stimulus onset unlike the tighter response durations in A1 and CM.

To compare at the level of the units in each field, we quantified response duration of each unit for each call by the half-peak width of the mean normalized PSTH (the temporal width at 50% of the peak; Figure 4A; see section Materials and Methods) for that unit across all pitch tokens of that call. The distributions of response duration for units in each field, for each call, are shown in Figure 4C. Overall, there were significant differences between the different populations [Kruskal–Wallis test, H(4) = 1071, *p* < 0.0001] and pair-wise comparisons showed the CL response duration was significantly longer than that in either A1 (Dunn's test, *p* < 0.0001) or CM (Dunn's test, *p* < 0.0001), and the CPB response duration was significantly longer than that in either A1 (Dunn's test, *p* < 0.0001) or CM (Dunn's test, *p* < 0.0001).

Thus, in response to vocalizations, CM and A1 responses were similarly fast and relatively short-lasting, whereas in CL and CPB, responses were slower and much longer-lasting. The PSTHs of CPB units appeared to be a slightly delayed version of the CL profile, with similar long-lasting response duration. Overall, response duration increased across fields as response onset was delayed.

### Spike Count and First Spike Latency as Coding Mechanisms for Vocalization Pitch

**Figure 5:**
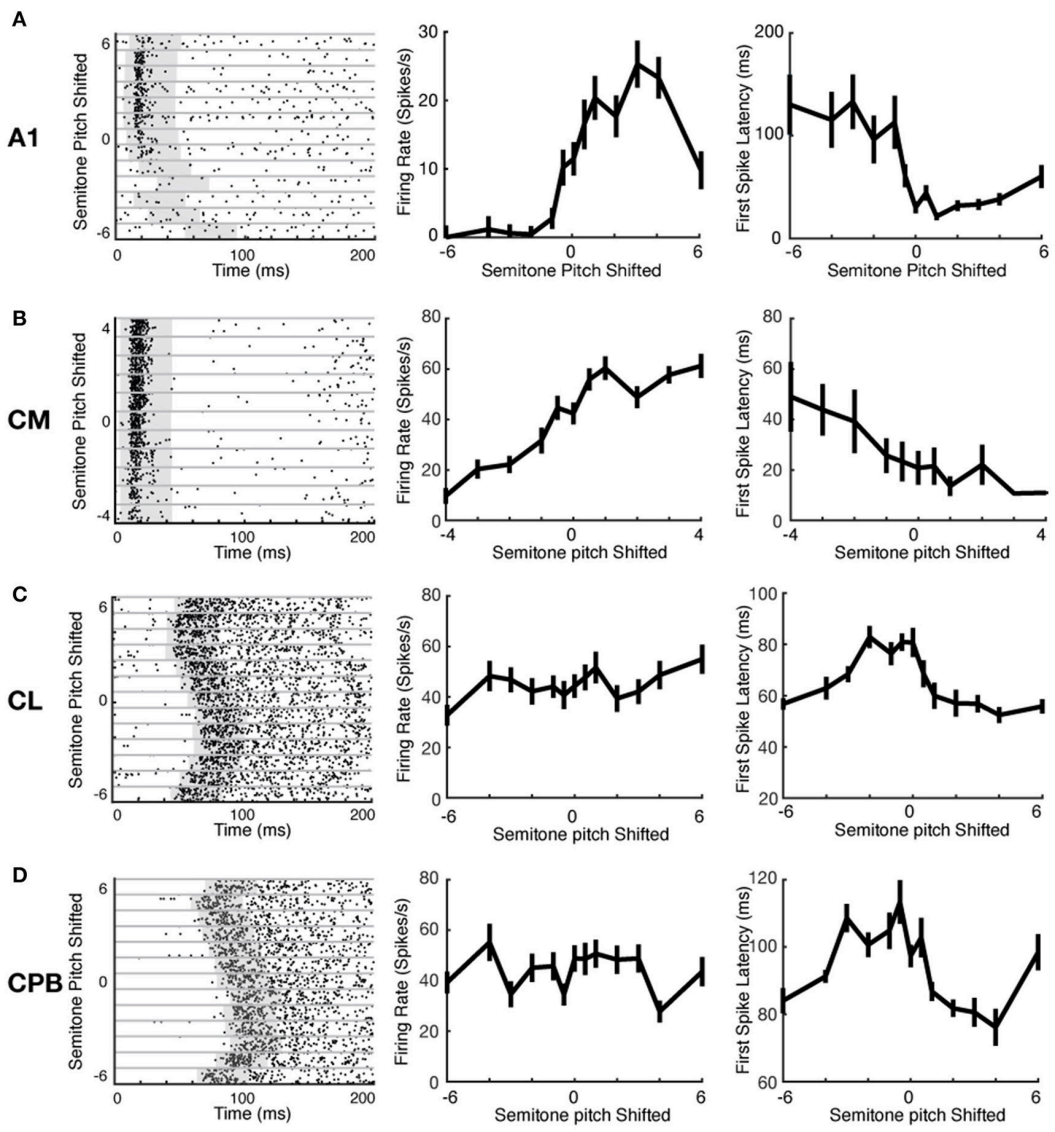
Example neuronal response raster plots and tuning curves to vocalization pitch changes. **(A–D)** Exemplar unit response to vocalization pitch changes. Label beside each row indicates the cortical areas from which the exemplar neuron was recorded. Left column: Exemplar unit response raster plots. Gray horizontal lines separate responses to different pitch token. Gray box indicates the time window used to calculate the response duration. Middle column: Tuning curves based on firing rate changes with pitch changes for the exemplar units for each area. Left column: Tuning curves based on changes in first spike-latency with pitch changes for the exemplar units for each area. A1 example was from response to Egg; CM example was form response to Egg; CL example was from response to Tsik; and CPB example was from response to Tsik.

Auditory cortical neurons can represent stimulus features via changes in spike-count or response latency (Bizley et al., 2010). We investigated if the different neuronal populations here used these two mechanisms differently to encode pitch changes. Figure 5 shows exemplar raster plots of responses of units from each cortical field to pitch changes (ordinate in left column panels of Figures 5A–D; abscissa in middle and right column panels of Figures 5A–D). These exemplars illustrate that, with changes in vocalization pitch, A1 and CM units could show marked variation in spike rates whereas any changes in spike-latency generally reflected a change from responsiveness to lack of response; in contrast, CL and CPB units showed marked variation in spike-latency with little or no change in spike rate. These effects are well-illustrated in the pitch tuning curves for the same units in Figure 5 (middle column: firing rate changes with pitch; right column: latency changes with pitch). The A1 unit exhibited non-monotonic pitch tuning curves to both spike-count and first spike-latency, and the CM unit showed monotonic pitch tuning with both measures. In these two exemplar units, as shown by the rasters, the change in latency was primarily due to a switch from pitches where responses were evoked to pitches where no reliable responses were evoked. In contrast, the CL and CPB units only exhibited significant pitch-associated modulation of first spike-latency, but not of spike-count.

We evaluated the tuning of each neuron to pitch variations in each call, using first spike-latency (as in Bizley et al., 2010) and mean spike-count as in our A1 study (Zhu et al., 2019). First spike-latency to each pitch token for a call was determined as the average time of the first spike after stimulus onset across trials. For spike-count, firing rate was the mean spike-count over a fixed 40 ms counting window varying in starting time, to capture responses in units where onset varied markedly with pitch (see Materials and Methods for details); window onset was set individually for each neuron for each stimulus (each pitch token for each call, gray box in raster plots in Figure 5), as follows. For each individual stimulus to each unit, the distribution of first spike-latency of responses across all trials of that stimulus was used to determine the 25th percentile of the first spike-latency. Then a 40 ms spike counting window was set to commence from 5 ms prior to that 25th percentile time point. This process was done separately for every unit's responses to every stimulus; individually set starting points for the fixed length window ensured that we did not miss spikes because of a later response onset time to a specific stimulus.

**Figure 6:**
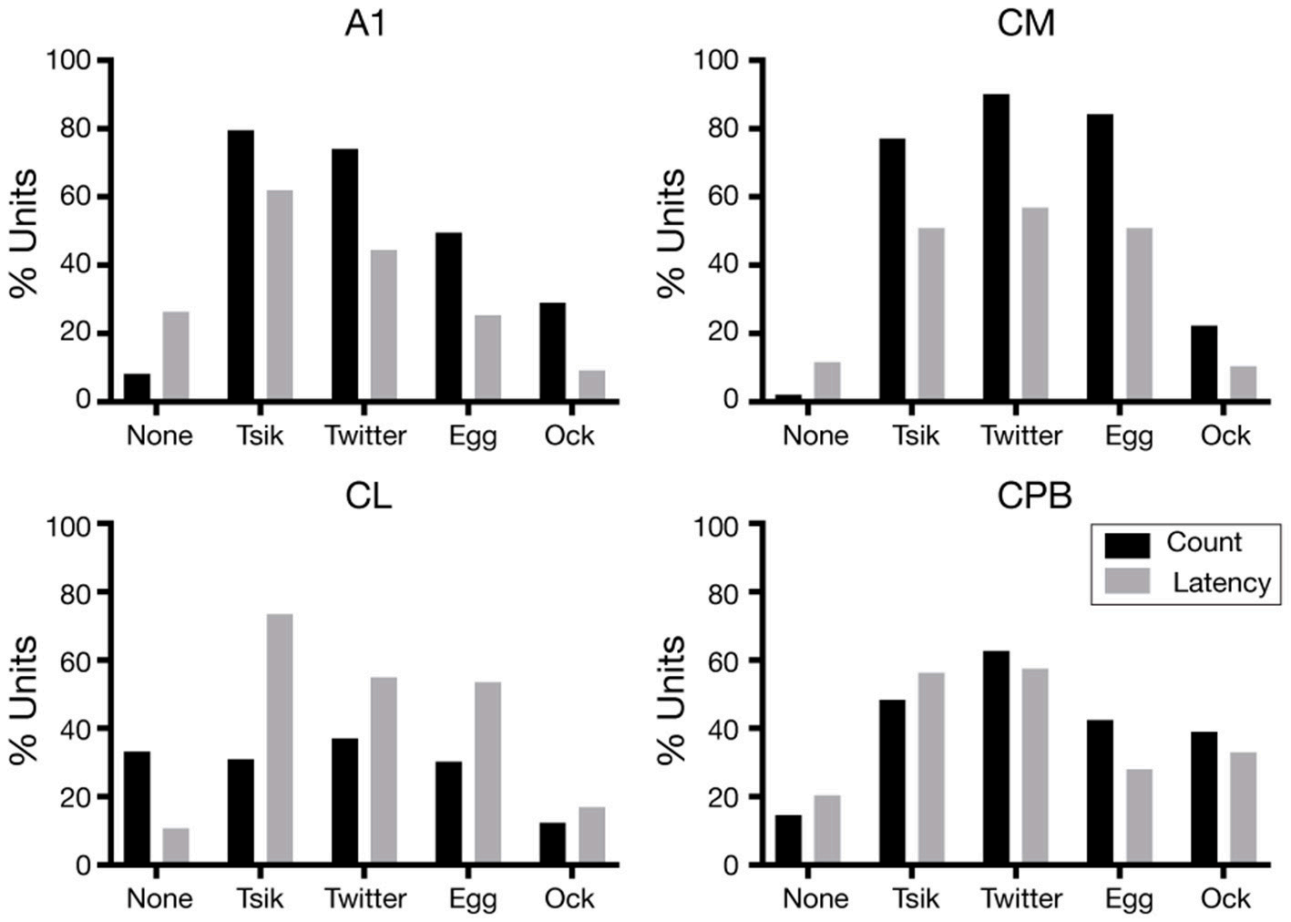
Proportions of pitch tuned units in the different neuronal populations, for the different calls. The proportions of units pitch tuned by spike-counts are shown in black and the proportions of units pitch tuned by first spike-latency variations are shown in gray.

For each unit the variations in first spike-latency (mean spike-count) with changes in pitch for each call were assessed by one-way ANOVA with Tukey's HSD test; units were classed as tuned to pitch changes by a measure if that metric was statistically significantly modulated by the pitch changes in the call. The proportions of units showing pitch tuning by the spike-count measure (including units with no tuning, “None”) (Figure 6 black bars), differed significantly across the four fields [Chi-Squared Test, $\chi_{\left(12\right)}^{2}$ = 276.4, *p* < 0.0001] as did the proportions of units showing pitch tuning by the spike-latency measure (Figure 6 gray bars) across the four areas [Chi-Squared Test, $\chi_{\left(12\right)}^{2}$ = 109.7, *p* < 0.0001].

We consider first the distributions in the two early-responding areas, A1 and CM. For A1, the overall distributions were significantly different between the two measures [$\chi_{\left(4\right)}^{2}$ = 73.4, *p* < 0.0001]; the latency measure gave a lower proportion of tuned units for all calls and a higher proportion of units non-tuned. Further, regardless of measure, many more A1 units were tuned to the two high *f*_*dom*_ calls *Tsik and Twitter* than to the two low *f*_*dom*_ calls *Egg and Ock* (Figure 6, A1). The CM tuning distributions resembled those for A1, again with a higher proportion of units showing tuning by spike-count compared to spike-latency [$\chi_{\left(4\right)}^{2}$ = 10.5, *p* < 0.05]. However, there was no reduction in the number of tuned CM units specifically for low *f*_*dom*_ calls: the proportions of units tuned by either measure to the *Egg* call were similar to the proportions of units tuned by either measure to the two high *f*_*dom*_ calls.

We next examined the distributions in the two late-responding areas, CL and CPB. Interestingly, CL had the lowest proportion of units showing spike-count-based tuning; >30% of CL units showed no tuning to any call by this measure, and this was higher than the proportion of tuned units for any call except the *Twitter*. Thus, in CL there was a significant difference between the overall distribution of tuning between the two measures [$\chi_{\left(4\right)}^{2}$ = 85.9, *p* < 0.0001] with more units showing pitch tuning by spike-latency across all calls, independent of the *f*_*dom*_ of the calls. In CPB, the difference in proportions of tuning by the two measures was less dramatic and, as for the early-responding cortical fields, more units showed spike-based tuning except for the *Tsik* call [overall distributions being significantly different: $\chi_{\left(4\right)}^{2}$ = 11.6, *p* < 0.05]; again, the differences between tuning proportions were independent of differences in *f*_*dom*_ between calls.

In summary, neurons in the four cortical areas could exhibit quite different temporal response patterns in encoding vocalizations. Response onset varied between fields with A1 and CM, having similar rapid response onsets and CL being slower still and CPB even slower. However, all four neuronal populations robustly retained the same unimodal-distributed response onset across the four vocalizations. Response duration differed between areas, in parallel with differences in the response onset. Auditory cortical neurons could code a fine range of variations in pitch through variations in spike-count or in first spike-latency, but the observation of differences in tuning proportions between spike-count and spike-latency measures suggests that different auditory cortical populations may differ in preferred coding mechanisms to encode vocalization pitch.

### Information Content in Different Coding Mechanisms of Different Neuronal Subpopulation

**Figure 7:**
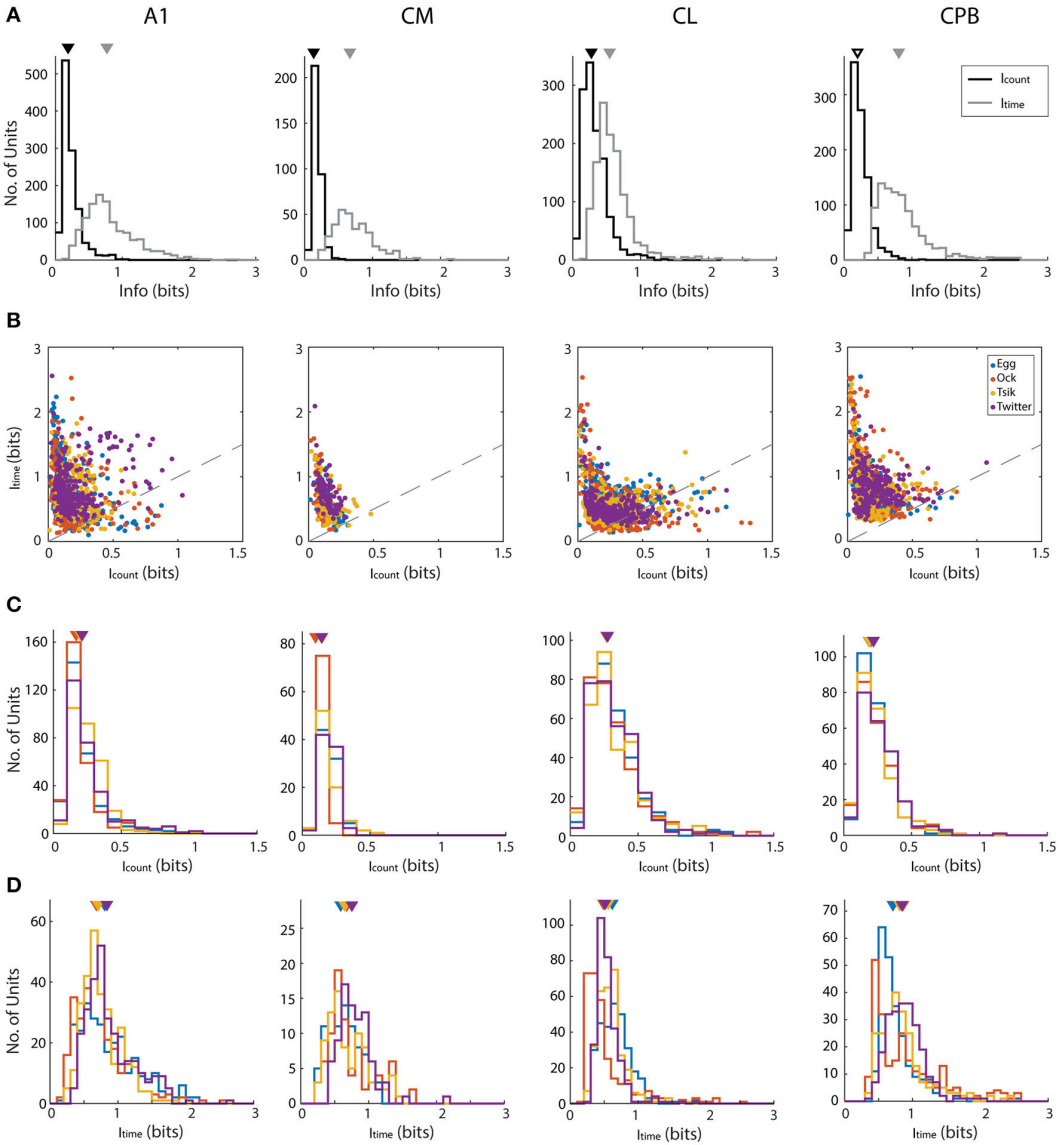
Mutual information about pitch in spike-count and first spike latency for different calls in different auditory cortex neuronal populations. **(A)** Distributions of information, calculated using conditioned entropy estimation, across all calls, in spike-count (black) and in first spike-latency (gray) of different auditory cortical populations (labels above each column of figures). In each panel the triangle indicates the mean of the distributions. Note that in all fields, first spike-latency carries much more information than does spike-count. **(B)** Scatter plots of information in spike-count vs. that in first spike-latency of individual units in different cortical fields. Different colors indicate different calls (see legend in row **B**, last panel) and the dashed gray line indicates the line of unity. For all calls in all fields, first spike-latency carries much more information than does spike-count. **(C,D)** Distribution of information content of spike-count **(C)** or of first spike-latency **(D)** for each of the four calls, in the four cortical fields. Triangles indicate the mean of each distribution and different colors indicate different calls (see legend in row **B**, last panel).

We determined if pitch-encoding by one measure was more informative than that by the other by determining how much information was carried in each measure, for the different fields; here we disregarded whether neurons were tuned to the call or not. We calculated information content in two ways: using the conditioned entropy estimation (see section Materials and Methods) or Victor's binless method (Victor, 2002) which controls the bias introduced when binning responses (Chase and Young, 2007). Both methods revealed the same pattern of bias in information content whereby first spike-latency contained more information than spike-count and hence we present only the information calculated using conditioned entropy (Figure 7).

For each call, we varied pitch by up to 11 different pitches and hence the maximum amount of information available is 3.5 bits [log_2_ (11)]. However, this theoretical maximum information value was considerably greater than the actual information (in bits) carried by neurons in any cortical field for any call. Thus, the largest information value by spike-count (I_count_) across calls and cortical fields was 1.329 from a unit in CL, while the largest information value by first spike-latency (I_time_) across all cortical field is 2.557 from a unit in A1. To generate descriptive data for each field, we pooled across all calls the information data by each measure separately for all units in each cortical field. This pooled data is shown in Figure 7A and shows that for all four fields and across all calls, I_count_ was almost always < 0.5 bits and that generally more information was carried by I_time_ for all four fields and all calls. The I_count_ differed significantly across the different populations [Kruskal–Wallis, K–W, test, H(4) = 349.7, *p* < 0.0001] and pair-wise comparisons showed significant differences between cortical fields and that CL units had the highest I_count_ and CM had the lowest I_count_. Pair-wise comparisons also revealed that the I_time_ also differed significantly between cortical fields [K–W test, H(4) = 458.4, *p* < 0.0001] and CPB units carried the highest information about pitch while CL units carried the lowest.

For each cortical field, we then compared for individual units the I_count_ against the I_time_ for different calls (Figure 7B). Across all fields and across all four calls, data were clearly shifted up from the unity line toward the I_time_ axis indicating the general trend of more information in first spike-latency compared to spike-count. This trend was more prominent in CM and CPB populations as almost all points were above the line of unity where A1 and CL had a few points below the unity line. Since there were more information in I_time_ compared to I_count_, we then compared the information carried in spike-count and first spike-latency separately for each call for each population, to examine if different cortical areas varied in information on pitch between the calls. The distributions of I_count_ and I_time_ for the different neuronal populations, for the different calls, is shown in Figure 7C (I_count_) and 7D (I_time_) and we first consider the distribution of I_count_ for the different calls for each population.

For A1, I_count_ (Figure 7C) was significantly different between calls [K–W test, H(4) = 56,41, *p* < 0.0001] due to differences between the high *f*_*dom*_ calls and the low *f*_*dom*_ calls (Dunn's multiple comparisons, *p* < 0.0005), with no significant differences between the pairs of high *f*_*dom*_ calls and the pairs of low *f*_*dom*_ calls. For CM, I_count_ was significantly higher in *Egg, Tsik* and *Twitter* compared to *Ock* [K–W test, H(4) = 44.9, *p* < 0.0001, Dunn's test, *p* < 0.0001]—note that, as shown in Figure 6, in this field tuning for the first three calls were similar and much higher than that for the *Ock* call, by either measure. For CL, I_count_ did not differ significantly between calls. For CPB, I_count_ differences only existed between the two high *f*_*dom*_ calls *Tsik* and *Twitter* calls [K–W test, H(4) = 9.16, *p* = 0.023, Dunn's test, *p* = 0.036].

With respect to I_time_ (Figure 7D), A1 units carried significantly higher information for *Twitter* and *Egg* calls compared to *Tsik* and *Ock* calls [K–W test, H(4) = 35.47, *p* < 0.0001, Dunn's test, *p* < 0.005]; this difference was clearly independent of *f*_*dom*_ since *Tsik* and *Twitter* are high *f*_*dom*_ calls and *Egg* and *Ock* are low *f*_*dom*_ calls. For CM units, I_time_ did not differ significantly between calls. For CL units, pair-wise comparisons revealed that I_time_ significantly differed between all call pairs [K–W test, H(4) = 134.4, *p* < 0.0001, Dunn's test, *p* < 0.05]; units had the highest I_time_ for the *Egg* call and the lowest I_time_ for the *Ock*. For CPB, units had the highest I_time_ for the *Twitter* call and the lowest I_time_ for the *Egg* call and the I_time_ for the *Egg* call differed significantly from those for the *Tsik* and *Twitter* calls [Kruskal–Wallis Test, H(4) = 36.35, *p* < 0.0001, Dunn's test, *p* < 0.05].

Overall, there was a bias of more information on pitch changes being contained in first spike-latency compared to spike-count. When only considering information measured with either code, we found significant differences across calls between different cortical fields and within a cortical field between different calls. The differences between fields in the information content of a given measure could reflect the differences in response patterns between cortical fields while the difference within fields may reflect their ability to utilize each code to signal pitch variations between different calls.

### Preference in Pitch Coding Mechanisms of Different Neuronal Subpopulations

The fact that the population mean of I_time_ always exceeded that of I_count_ in each field suggested that if pitch discriminability governed by each measure may also differ between fields. We generated receiver operating curves for pitch discriminability for each call (Zhu et al., 2019) based on spike-count and on first spike-latency and compared the area under the curve (aROC) between two end conditions—the preferred pitch and null pitch for that call. For spike-count, the preferred pitch for a call was the pitch token with the highest spike-count while the null pitch was the one with the lowest. For first spike-latency, the preferred pitch was that with the shortest mean first spike-latency while the null pitch was the one with the longest. Note that while the preferred pitch and the null pitch may not be the same for the two measures (c.f, Figure 5), that difference is not material for the comparison of discriminability between the two end conditions, i.e., how well-separated the response distributions were for two end conditions of pitch.

**Figure 8:**
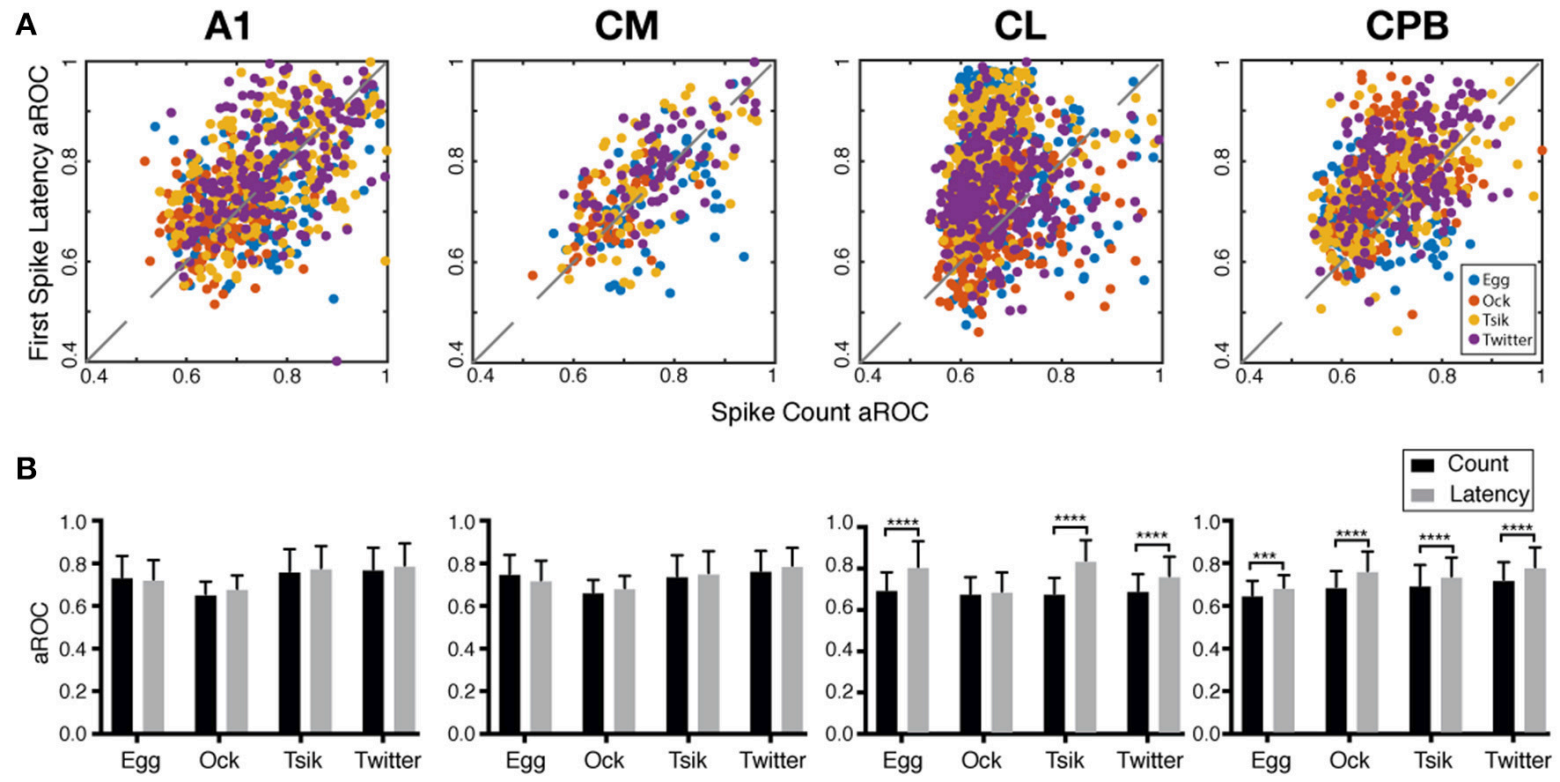
Area under the receiver operating characteristic curve (aROC) by spike-count and first spike-latency for different subpopulations. **(A)** Comparison of aROC, by each of the two measures, between the preferred pitch and the null pitch for each call (different colors indicate different calls). For spike-count, the preferred pitch for a call was the pitch token with the highest spike-count while the null pitch was that with the lowest. For first spike-latency, the preferred pitch was that with the shortest mean first spike-latency while the null pitch was that with the longest latency. **(B)** Mean aROC and standard deviation for the different calls in the different cortical fields. Black bar represents aROC by spike-count. Gray bar represents aROC by first spike-latency. ^***^p < *0.005;* ^****^p < *0.001*.

Figure 8A plots the aROC value by the spike-count measure for an individual unit against that for the same unit by the spike-latency measure. For all calls, aROC values in A1 and CM were scattered along the unity line but both CL and CPB populations showed a scatter skewed toward spike-latency, suggesting differential preferences for coding mechanism across the neuronal populations regardless of call. Figure 8B compares the mean aROC scores for each of the two measures for different calls within each subpopulation. There were no differences between the two measures for all calls for A1 and CM neurons. However, for CL, aROC values significantly differed between the two measures [Two-way ANOVA, call type: *F*_(3, 2, 206)_ = 77.88, *p* < 0.0001; coding measure: *F*_(1, 2, 206)_ = 513.5, *p* < 0.0001], for all calls tested (Sidak's multiple comparisons, *Egg*: *p* < 0.0001; *Ock*: non-significant; *Tsik*: *p* < 0.0001; *Twitter*: *p* < 0.0001). The aROC scores for the CPB population also significantly differed between measures [Two-way ANOVA, call type: *F*_(3, 1, 394)_ = 66.95, *p* < 0.0001; coding measure: *F*_(1, 1, 394)_ = 146.8, *p* < 0.0001; interaction: *F*_(3, 1, 394)_ = 4.053, *p* = 0.0070], for all calls tested; Sidak's multiple comparisons, *Egg*: Mean Diff. = 0.035, *p* = 0.0003; *Ock*: Mean Diff. = 0.076, *p* < 0.0001; *Tsik*: Mean Diff. = 0.044, *p* < 0.0001; *Twitter*: Mean Diff. = 0.059, *p* < 0.0001.

Thus, in early-responding cortical fields, the pitch discriminability between two end conditions was similar by spike-count and spike-latency measures but in later responding cortical fields, spike-latency gave the higher pitch discriminability between the end conditions.

### Pitch Coding Is Closely Related to Temporal Response Patterns and Integration Window and Population Size Affect Preferred Coding Mechanism

To probe these differential preferences in coding mechanisms, we developed and tested linear decoders which used one of the two measures to assign responses to the specific stimulus variable (in this case, pitch). Due to the very different temporal response patterns amongst the different populations, we needed to first establish how differences in spiking activity across time affected decoder performance. We randomly selected a sample of pitch-tuned units from each field and, for each call, examined the spike-count-based decoder performance and the first spike-latency based decoder performance in a series of integration windows. To ensure sample size was not a confounder that affected decoder performance, for each call we used a fixed sample size for both the spike-count-based decoder and the first spike-latency-based decoder. The smallest sample size was from CM, and to match with it, a sample size of 40 units was used for *Tsik, Twitter*, and *Egg* for both decoders and a sample size of 9 was used for *Ock*. Decoding was done separately using units pitch-tuned by the spike-count measure or units pitch-tuned by the spike-latency measure.

**Figure 9:**
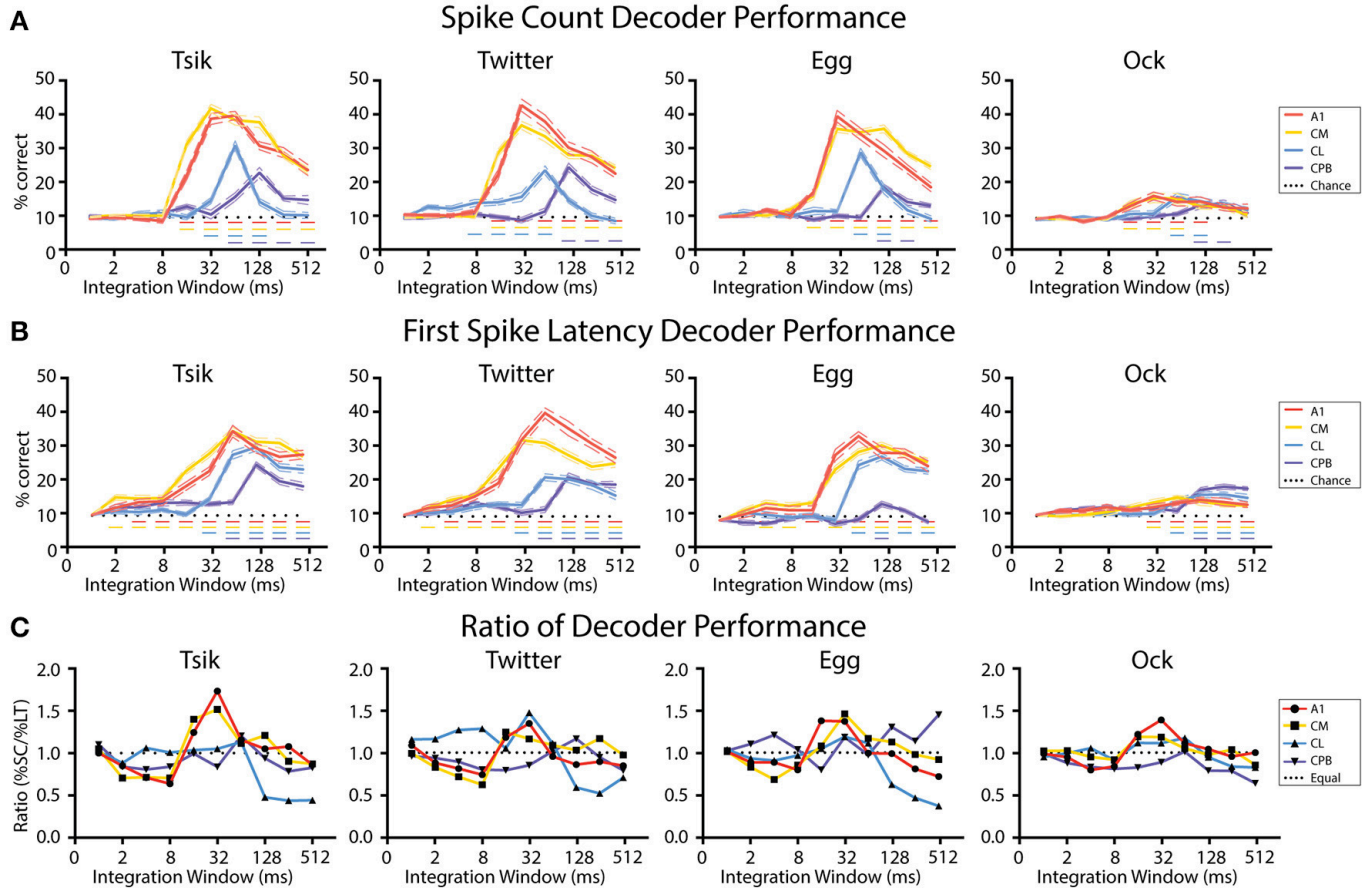
Performance of linear decoders for pitch classification as a function of integration window, for different auditory cortical populations. **(A)** Performance of a spike-count-based decoder for pitch classification, as a function of integration window. **(B)** Performance of a first spike-latency-based decoder for pitch classification, as a function of integration window. **(C)** Comparison of the performance of the two decoders, determined as the ratio of their performance at each integration window. Dotted line indicates a performance ratio of 1:1 indicating equal performance of the two decoders, while values >1 indicate better performance of the spike-count-based decoder. In **(A–C)** different colors indicates data from different cortical populations. In **(A,B)** chance level (1/11) is denoted by the dotted line; Dashed colored line indicates performance that is statistically significant higher than chance (*p* < 0.05); the color of the dashed line corresponds to the different neuronal populations.

Figure 9A shows the effect of integration window on decoder performance for units pitch-tuned by spike-count. For all cases, the performance curve resembled the time course of the spiking activity of each population: early-responding populations (A1, CM; c.f., Figure 4) reached peak decoder performance in an earlier integration window (typically 32 ms window) than did the late responding populations (CL, CPB; CL—integration window of at least 64 ms; CPB population-−128 ms). Then, for each window, we compared decoder performance against chance performance generated by training and testing the decoder with trials from random pitch tokens of each call. For all four populations, as integration window increased beyond that for peak performance, decoder performance declined. Despite this, for A1 and CM, decoder performance for all calls except *Ock* never dropped to chance level even with an integration window of 512 ms which well-exceeded the response duration (see Figure 4) of A1 and CM units [Two-way ANOVA, Tsik: cortical field: *F*_(4, 950)_ = 302.9, *p* < 0.0001; integration window: *F*_(9, 950)_ = 160.6, *p* < 0.0001; interaction: *F*_(36, 950)_ = 33.83, *p* < 0.0001; Twitter: cortical field: *F*_(4, 950)_ = 30.37, *P* < 0.0001; integration window: *F*_(9, 950)_ = 108.8, *p* < 0.0001; interaction: *F*_(36, 950)_ = 33.83, *p* < 0.0001; Egg: cortical field: *F*_(4, 950)_ = 305.8, *p* < 0 0.0001; integration window: *F*_(9, 950)_ = 154.2, *p* < 0.0001; interaction: *F*_(36, 950)_ = 37.5, *p* < 0.0001; Ock: cortical field: *F*_(4, 950)_ = 10.03, *p* < 0.0001; integration window: *F*_(9, 950)_ = 11.86, *p* < 0.0001; interaction: *F*_(36, 950)_ = 2.1, *p* = 0.0002; with Dunnett's multiple comparisons, *p* < 0.05 indicated by dashed horizontal line]. For CL, decoder performance was also affected by integration window, dropping to chance level when integrating spikes for longer than 128 ms for all calls. Decoder performance for CPB was much less affected by integration window and, except for *Egg* and *Ock*, increasing it beyond the time after achieving peak performance did not significantly affect performance. The sensitivity of the CL population decoder to integration window suggests that spike-count might not be a reliable coding mechanism for pitch in CL and an alternative coding mechanism may be required.

Figure 9B shows the effect of integration window on decoder performance using units pitch-tuned by first spike-latency. A very similar pattern was observed as with spike-count: decoder performance peak for different fields was well-correlated with response temporal pattern and the early-responding A1 and CM populations achieved their peak performance at about 32–64 ms while the later responding CL population peaked at about 64–128 ms and the even later CPB population peaked at 128 ms [Two-way ANOVA, Tsik: cortical field: *F*_(4, 950)_ = 223.9, *p* < 0.0001; integration window: *F*_(9, 950)_ = 131.1, *p* < 0.0001; interaction: *F*_(36, 950)_ = 15.8, *p* < 0.0001; Twitter: cortical field: *F*_(4, 950)_ = 290.3, *p* < 0.0001; integration window: *F*_(9, 950)_ = 105.8, *p* < 0.0001; interaction: *F*_(36, 950)_ = 20.91, *p* < 0.0001; Egg: cortical field: *F*_(4, 950)_ = 246.7, *p* < 0.0001; integration window: *F*_(9, 950)_ = 115.8, *p* < 0.0001; interaction: *F*_(36, 950)_ = 19.6, *p* < 0.0001; Ock: cortical field: *F*_(4, 950)_ = 30.01, *p* < 0.0001; integration window: *F*_(9, 950)_ = 22.19, *p* < 0.0001; interaction: *F*_(36, 950)_ = 3.85, *p* < 0.0001; with Dunnett's multiple comparisons, *p* < 0.05 indicated by dashed horizontal line]. Again, for all populations, decoder performance gradually dropped as integration window increased beyond that at which peak performance occurred. Interestingly, except for decoding pitch of *Egg* in CPB, decoder performance always remained higher than chance even when the integration window increased beyond the time when peak performance was achieved.

In summary, these results show that decoder performance closely followed the temporal response pattern of the population. Unsurprisingly, it was also sensitive to integration window size-increasing integration window beyond the response duration of a population should result in a decrease in decoder performance as a consequence of including spikes unrelated to the stimulus. This effect was especially detrimental for the spike-count decoder for CL, where responses could drop below chance.

We then investigated if one code was more reliable with respect to integration window by calculating the ratio between decoder performance by spike-count and by first spike-latency. As shown in Figure 9C, the preference for coding mechanisms varied between different neuronal populations in line with their response onset profile. For the early-responding populations A1 and CM, for all calls there was initially better performance (ratios < 1.0) with the first spike-latency decoder and then, as integration window reached 8 ms and both decoders started improving toward their peak performance, there was a switch to the spike code decoder (ratios > 1.0) outperforming first spike-latency. Then, once peak performance had been reached for both decoders, as integration window kept lengthening, both decoders performed equally well for A1 and CM where the performance ratio fluctuating around 1 for all calls.

For CL, the spike-count decoder either had a similar performance or slightly outperformed the first spike-latency decoder before both reached their peak performance and then, for all calls, the first spike-latency decoder became the better coding mechanism as the integration window kept lengthening. For CPB, a consistent pattern was observed that first spike-latency decoder performs better under most integration window except for transient better performance of spike-count decoder at the window when peak performance was reached for all calls expect *Egg*, of which the decoder performance ratio fluctuating around 1.

In summary, for A1 and CM, for integration windows ≥16 ms, the spike-count decoder either performed equal to or better than the spike-latency-based decoder. In contrast, for the late responding CL and CPB populations, the performance of spike-count-based decoder was greatly affected by integration window and overall the first spike-latency-based decoder performed better. These observations suggest different fields may have different preferred mechanisms for encoding vocalization pitch, determined by the integration window.

**Figure 10:**
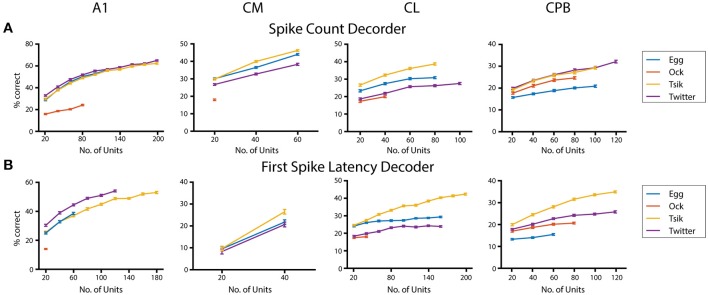
Effects of population size on decoder performance. **(A)** Spike-count based decoder performance with different sample size. **(B)** First spike-latency based decoder performance with different sample size. Different color indicates different call tested.

Finally, given that population size appears to affect neurometric performance in auditory cortex (Bizley et al., 2010), we examined its effect on performance of the pitch decoders (Figure 10) using the integration window that gave peak decoder performance by each of the two measures for each call, in each field; again we used only units that showed pitch tuning by either measure even if the two groups did not contain all the same units. For all calls, we tested each population with a range of sample sizes increasing in 20-unit steps. The maximum number of units tested was limited by our data size and the number of tuned units in that population. For the spike-count based decoder (Figure 10A), all neuronal populations showed significant population size effects for each call (One-way ANOVA, *p* all < 0.0001). Testing the effect of population size on the performance of the first spike-latency based decoder with the same series of testing sample size (Figure 10B) also showed a significant improvement in decoder performance with increasing sample size (One-way ANOVA, *p* all < 0.0001). For the different populations, best performance by either measure varied between calls but, for a fixed sample size, best performance was usually observed for the high *f*_*dom*_ calls (either *Tsik* or *Twitter*) and worse for the low *f*_*dom*_ call (mostly *Ock* in all fields except for *Egg* for CPB). Limited to the size of our dataset, we failed to estimate the maximum decoder performance to each call. We also compared the decoder performance at a common sample size within each subpopulation between calls and revealed significant differences in performance between calls suggesting the ability of encode pitch changes of different calls was different within subpopulations.

## Discussion

In this study we investigated the mechanisms for neuronal encoding of variations in the pitch of natural vocalizations in core (A1) and a set of caudal belt (caudal medial, CM, and caudal lateral, CL) and CPB auditory cortex fields. These fields differ in basic neuronal response characteristics including pure tone tuning bandwidths (determined from the FRAs) and temporal response features like latency and duration, as also reported previously (Recanzone, 2000; Hackett, 2011). We found that, as in Macaque monkeys (Recanzone, 2008; Russ et al., 2008; Kusmierek and Rauschecker, 2009), neurons in these core and non-core fields of marmoset auditory cortex were responsive and could differentiate between conspecific vocalizations but we also demonstrated, for the first time, that neurons in these fields showed differences in temporal response patterns to vocalizations. In general, the neurons in CL and CPB, with significantly larger mean FRA bandwidths than in A1 and CM, were slower to respond and had longer-lasting responses to vocalizations than the narrower-bandwidth and faster-responding and shorter-lasting A1 and CM neurons. We also found that the broadening of tuning bandwidth in the CL and CPB fields, relative to the A1 and CM fields, did not lead to lack of pitch sensitivity and that neurons in all four fields could encode fine scale pitch changes in individual vocalizations.

Our experiments were conducted under anesthesia as only the anesthetized condition allows the collection of the large volume of data we needed to compare between cortical fields, the collection across multiple fields in the one animal, and the post-mortem histological examination to confirm the location of the recording sites in different fields identified by histology as an independent confirmation to the physiological topographic (cochleotopic) organization. We acknowledge that responses in the anesthetized condition may differ from those in the awake condition but note that we have shown in previous studies using exactly the same anesthesia as used here (Rajan et al., 2013; Lui et al., 2015) that this anesthetic regime has minimal effects on neuronal responses compared to what has been seen in studies in awake animals (Wang et al., 2005) using complex signals, and largely preserves the neuronal response to complex stimuli such as the vocalizations we used here. In fact, we found high and dense levels of activity in the belt fields CL and PB, presenting a further argument that our anesthetic does not suppress activity in high-order fields in auditory cortex which are otherwise susceptible to anesthetic suppression with other agents. Nevertheless, it would be interesting to further investigate the encoding of vocalization pitch and the discriminability to vocalization pitch in awake behaving animals if conditions could be created to obtain the same wealth of data across multiple fields as we have achieved in this study.

We used one exemplar of each of four natural vocalizations recorded from monkeys not included in this study, and applied pitch changes to these sounds by carefully varying their frequency spectrum without altering their temporal structure. The frequency modulations we applied mimic the range of naturally occurring *f*_0_ differences among individual conspecifics (Agamaite et al., 2015). It might be argued that for generalization of our results, we could have also tested more exemplars of each sound; however, other exemplars may likely vary from the one we used but this be due to factors such as recording equipment characteristics, variations in ambient background noise levels during recordings in natural settings or in cages, differences in animal weight, degree of distress of animals, etc., none of which has any bearing on the encoding of pitch *per se*. Our protocol is ideally designed to evaluate the encoding of pitch of natural vocalizations.

### Coding for Vocalization Pitch in Different Auditory Cortical Fields

We have previously shown that A1 neurons can use firing rate to encode the pitch of vocalizations (Zhu et al., 2019) or the spatial location of vocalizations (Lui et al., 2015), consistent with studies, across different auditory fields, showing the use of spike-count for multi-dimensional representation of sound features (Panzeri et al., 2010; Walker et al., 2011). An alternative strategy is to use spike-latency which has been suggested to carry as much, and in some instances even more, information than firing rate, especially when referenced to the onset of responses in the population (Chase and Young, 2007; Gollisch and Meister, 2008; Panzeri et al., 2010). In our present study, neurons in the four different cortical fields could use first spike-latency or spike-count to encode pitch changes, but differed in the preferred coding mechanisms, consistent with functional differences between the fields. All populations had a high percentage of neurons with pitch sensitivity if both spike-count and first spike-latency measures were taken into account. In CL, the reduced number of cells with pitch sensitivity when using only spike-count as a measure was increased to 80% by using first spike-latency, and more than 80% of CPB units showed sensitivity to pitch changes of at least one call using either measure.

The putative use of first spike-latency as an auditory coding mechanism has been demonstrated for other sound features. At different processing levels up to cortex, first spike-latency may carry information about stimulus frequency (Heil, 1997; Tan et al., 2008; Zhou et al., 2012); stimulus location can be encoded in spike-latency changes, in neurons from brainstem to auditory cortex (Eggermont, 1998; Furukawa and Middlebrooks, 2002; Stecker and Middlebrooks, 2003; Chase and Young, 2008); first spike-latency can contain information about sound identity (Russ et al., 2008; Bizley et al., 2010); relative population latency can encode stimulus periodicity (which underpins pitch discrimination for such sounds) as efficiently as spike-count (Bizley et al., 2010); and combined with rate coding, latency coding adds important information on stimulus periodicity to improve population encoding of this sound feature (Bizley et al., 2010). One issue about using first spike-latency as a reliable coding mechanism is whether changes in first spike-latency are sufficiently large to allow decoding of stimulus identity (Zhou et al., 2012): we found that first spike latency could reliably encode pitch and may outperform spike-count code depending on neuronal populations and integration windows. Another concern arises from the lack of knowledge in the brain of the onset of the external stimulus (Heil, 2004). Previous studies have addressed this concern by investigating the relative latency to one or more reference neurons in a simultaneously recorded population and showed that relative latency could be a reliable neural code (Jenison, 2001; Stecker and Middlebrooks, 2003; Chase and Young, 2007; Bizley et al., 2010). We recorded responses across different cortical fields from multiple animals and multiple recording sessions, and hence compared the encoding of vocalization pitch with reference to stimulus onset, a consistent reference point across all recording sessions and animals.

Caudal auditory cortical belt fields are selective for spatial information (Rauschecker and Tian, 2000; Tian et al., 2001), but neurons in these regions are also selective to conspecific vocalizations (Tian et al., 2001; Recanzone, 2008). One way to achieve multiplex representations is by using different aspects of the spike train such as spike-count over different windows or first spike-latency to represent different features of the sound (Walker et al., 2011). We did observe significant differences in pitch sensitivity distribution between the two measures for all populations, with most prominent differences in A1, CM, and CL: the first two showed a larger proportion of pitch sensitive neurons by spike-count measures while CL showed the opposite. We speculate that neurons in the putative location-processing field CL may use first spike-latency to represent sound identity and use spike-count to represent sound location.

### Decoding Vocalization Pitch Using Spike Count or Spike Latency in Different Auditory Cortical Fields

In all fields and for all calls stimulus related information in first spike-latency was always higher than that in spike-count, in line with a study on information content of these two measures in retinal ganglion cells (Gollisch and Meister, 2008). An alternative information estimation approach, the Victor's binless method, confirmed that our observation of more information in first spike-latency was not due to bias from binning of responses. For either measure, information content differed between calls.

To further investigate coding mechanisms in different cortical fields for vocalization pitch, we also developed linear decoders using the two neural codes. We found that calls with larger information content tended to have better decoder performance, i.e., differences in information between calls may contribute to differences in the populations' ability to decode pitch changes. For example, for CPB, Egg had the lowest mean information in first spike-latency as well as the lowest decoder performance. This relationship was not perfect and there was some discrepancy between information content and decoder performance, possibly caused by the inclusion of both pitch-tuned and un-tuned units for the information estimation analyses but only pitch-tuned units for the decoding analysis.

When decoding the full range of pitch variations, we found that the preference for coding mechanism could be highly dependent on integration window and that the neurometric performance of both measures were closely correlated with the response time course of the population under consideration. Using the spike-count based mechanism, early-responding populations A1 and CM reached peak decoding performance quickly, after accumulating spikes for about 32 ms (similar to the value for periodicity encoding seen in a previous study in ferrets, Bizley et al., 2010), compared to the late responding populations CL and CPB. Optimal performance of latency mechanism was not achieved until 64 ms for A1 and CM neurons but the latency code showed better performance than spike-count code in A1 and CM in the early period up to 16 ms post-stimulus onset (see Figure 9C), in line with proposals that first spike-latency can lead to fast stimulus discrimination (Johansson and Birznieks, 2004). For CL and CPB neurons, the optimal performance of the spike-count code and the latency code was achieved around the same time and the performance of the latency code was better than the spike-count code beyond peak performance, suggesting that in these caudal cortical areas, stimulus identity is better represented by first spike-latency. Neurons in caudal belt fields represent sound source location with modulation of their spike-count (Remington and Wang, 2018). Our results suggest these neurons also remain sensitive to sound identity by utilizing spike-latency to represent spectral differences when sound source location is kept constant as we have. In later reports we will examine how these spectral differences affect location coding in putative location-coding caudal auditory cortical neurons.

Increasing integration window beyond the peak had a significant detrimental effect only on spike-count-based decoding of pitch in delayed-responding CL neurons but not in early-responding A1 and CM neurons or in the delayed-responding CPB neurons. Thus, the brief response duration of the early-responding populations did not limit their ability to encode response identity and offers stable and rapid neuronal activity for the brain to read out stimulus identity. For CL, spike-count appears to be an unreliable coding mechanism to represent vocalization pitch. With respect to preferred coding mechanism, for A1 and CM, vocalization pitch variations induced large and reliable changes in spike-count and first spike-latency while, in CL and CPB, changes in first spike-latency were more profound and reliable.

In concord with previous observations (Bizley et al., 2010) expanding population size could improve decoder performance. Since we observed a mixed representation of pitch selectivity by either spike-count or spike-latency across all subpopulations, for a fixed population size, the measure that gives a larger proportion of tuning could contribute more to perceptual discrimination. However, we were not able to estimate the optimal population size for encoding pitch differences due to the lack of marmoset psychometric data for discriminating pitch differences of the conspecific vocalizations used here. Nevertheless, our data suggested that neurons across different auditory field possess the ability to discriminate fine pitch differences in vocalizations.

### Hierarchical Segregation of Auditory Cortex

Our study does not provide support for previous hierarchical parcellation of auditory cortex into core and belt fields varying in order of information flow and in functional specialization (Hackett, 2011).

With respect to the classification of A1 as core and CM as belt we found no evidence supporting a simple hierarchy of information flow between these fields. Response latencies, response durations and response temporal profiles to vocalizations in CM neurons were similar to those in A1 (Recanzone, 2000). The bandwidth of the pure tone FRA is another major response parameter reported to differ between auditory fields, with increasing bandwidth along the core-belt axis (Hackett, 2011). Again, we did not find a simple hierarchy between A1 and CM: bandwidths were narrowest in A1 but not different from those in CM. Finally, neurons in CM largely behaved like A1 neurons in their pitch discriminability and preference for pitch coding mechanisms. These striking similarities between these two areas raise questions about whether CM can truly be considered as a secondary area. A previous study in macaque showed that lesion of A1 diminished responses in CM (Rauschecker et al., 1997) but that study included the lateral portion of belt in front of A1 as part of CM and did not specify the response onset time of those CM neurons. It is possible that the lack of response after lesion occurred in CL rather than CM. Our data suggest that CM and A1 may be parallel areas that process auditory information concurrently.

We also did not find evidence to support classifying CM and CL as being at the same (second) hierarchical level. CL neurons had significantly longer response latencies and longer-lasting response profiles compared to the CM. In fact, the temporal parameters of responses in CL (belt) were only slightly earlier, faster and shorter-lasting than responses in the parabelt field, which had the longest response latency (c.f., Hackett, 2011). Bandwidths were very much broader in CL and CPB than in CM and A1, again speaking against a classification of CM and CL as being at the same level. Finally, the mean CL bandwidth was significantly broader than for the CPB field.

Overall, our data suggest that a reappraisal of the hierarchy of auditory cortex may be warranted and we will address this issue in later reports.

## Data Availability Statement

The datasets supporting the findings of this manuscript will be made available by the authors, with no reservation, to any qualified researchers upon request.

## Author Contributions

SZ and RR designed the experiments and wrote the manuscript. SZ, BA, and RR performed the electrophysiology experiment. MR and LL performed the surgical preparation. AS generated the stimuli and stimuli presentation system. SZ performed the data analysis. MR edited the manuscript.

### Conflict of Interest Statement

The authors declare that the research was conducted in the absence of any commercial or financial relationships that could be construed as a potential conflict of interest.

## Abbreviations

A1: primary auditory cortex
CM: caudal medial belt
CL: caudal lateral belt
CPB: caudal parabelt
R: rostral field
CB: caudal belt
ML: medial lateral belt
FRA: frequency response areas
*f*_0_: fundamental frequency
*f*_*dom*_: dominant frequency
aROC: area under the receiver operating characteristics.
